# Image-based identification and DEA-based optimization modeling of antibiotic packaging using unsupervised learning techniques

**DOI:** 10.1371/journal.pone.0354277

**Published:** 2026-07-27

**Authors:** Phakdee Sukpornsawan, Yutthapoom Meepradist, Titinun Auamnoy, Ureerat Suksawatchon, Somchart Chokchaitam, Suthabordee Muongmee

**Affiliations:** 1 Faculty of Pharmaceutical Sciences, Burapha University, Chonburi, Thailand; 2 Faculty of Informatics, Burapha University, Chonburi, Thailand; 3 Faculty of Engineering, Thammasat University, Pathum Thani, Thailand; University of the Philippines Diliman, PHILIPPINES

## Abstract

**Background:**

Ensuring medication safety requires accurate identification of antibiotic packaging, especially within pharmacy automation and dispensing systems. Advanced imaging and machine learning offer novel avenues for physical package recognition.

**Objective:**

To investigate visual and textual features of antibiotic packages and evaluate their relationship with identification outcomes using unsupervised learning and efficiency-based analysis.

**Methods:**

Thirty-six antibiotic formulations from Thailand (2016–2021) were analyzed using binary imaging, entropy metrics, packaging area ratio (PAR), and optical character recognition (OCR). K-means clustering was applied to segment package groups, and data envelopment analysis (DEA) was used to assess relative efficiency without assuming predefined functional relationships between inputs and outputs.

**Results:**

Nine distinct image clusters were identified. Packages with mid-range entropy (7.1–7.5) and PAR (1.2–1.45) were associated with higher identification consistency. OCR text confidence influenced identification outcomes. DEA identified clusters with relatively efficient input–output configurations.

**Conclusion:**

Integrating image-derived metrics and OCR-based features supports automated antibiotic package identification. This framework provides a structured approach for evaluating packaging characteristics in pharmacy workflows.

## 1. Introduction

Medication safety remains a persistent challenge in contemporary healthcare systems, particularly as pharmacy workflows increasingly rely on automation, high-throughput dispensing, and distributed supply chains [1,2]. Although digital technologies have improved prescription processing and inventory management, verification tasks at the point of dispensing often still depend on rapid visual assessment of pharmaceutical products. This reliance on visual inspection introduces vulnerability within automated workflows, especially in settings characterized by high workload, heterogeneous product sourcing, and non-standardized packaging designs [3].

Antibiotics represent a critical medication category within this context. They are among the most frequently dispensed medicines worldwide and are closely associated with patient safety, antimicrobial stewardship, and the global burden of antimicrobial resistance (AMR) [1–3]. In many low- and middle-income countries, including Thailand, antibiotic markets are dominated by generic products from multiple manufacturers, often displaying visually similar packaging with variable print quality, reflective materials, and inconsistent layout conventions [4–6]. Such characteristics increase the likelihood of look-alike packaging confusion and complicate digital verification processes in pharmacy practice.

Recent advances in computer vision and artificial intelligence have enabled automated identification of pharmaceutical products based on visual characteristics. Prior studies have largely focused on pill-level recognition using features such as shape, color, imprint, and surface texture [7,8]. While these approaches demonstrate promising performance under controlled conditions, their applicability is limited in routine pharmacy workflows, where medicines are commonly handled and verified in their packaged form rather than as isolated dosage units. Packaging-level identification therefore represents a complementary but less explored domain within medication safety informatics.

Optical character recognition (OCR) has been widely adopted to extract alphanumeric information from documents and labels and has been applied in pharmaceutical contexts such as batch code reading and regulatory verification [9,10–13]. However, OCR performance is sensitive to surface reflectivity, background noise, and print quality—factors that vary substantially across antibiotic packaging types. When applied in isolation, OCR may exhibit unstable performance in heterogeneous packaging environments, highlighting the need for analytical frameworks that integrate textual confidence with physical packaging characteristics.

Unsupervised learning methods, including clustering techniques, offer a data-driven means of exploring latent structure in heterogeneous image datasets without reliance on predefined labels. Such approaches are particularly suitable when ground truth labels are incomplete or inconsistently defined [14–16]. However, clustering alone does not provide a principled mechanism for evaluating how different feature configurations support identification outcomes. Consequently, a methodological gap remains between exploratory image analysis and decision-oriented evaluation in packaging-level identification studies.

Data Envelopment Analysis (DEA), a non-parametric optimization method traditionally used for efficiency benchmarking, offers a potential approach for addressing this gap. DEA enables comparative efficiency assessment without assuming predefined functional relationships between inputs and outputs, meaning that no specific mathematical form is imposed on how input variables are transformed into outputs. Although DEA has been applied in healthcare operations and pharmaceutical manufacturing, its use as an evaluation layer within image-based packaging identification frameworks remains limited.

Key image-based metrics considered in this study include entropy as a descriptor of surface texture, packaging area ratio (PAR) as a spatial normalization measure, and OCR-derived confidence metrics reflecting text readability.

Against this background, the present study examines how physical and textual features of antibiotic packaging interact to influence automated identification outcomes within a unified analytical framework. Rather than proposing a supervised classification or prediction model, this work adopts a packaging-level informatics perspective that integrates image-derived descriptors, OCR-based confidence metrics, unsupervised clustering, and DEA-based efficiency evaluation.

Specifically, this study analyzes 36 commonly used antibiotic packages in Thailand, characterizing their packaging area ratio, entropy-based texture descriptors, and OCR confidence measures. K-means clustering is employed to explore latent structural groupings, while DEA is applied as a comparative evaluation layer to benchmark feature configurations under fixed analytical assumptions.

The contribution of this study is threefold. First, it shifts the focus of automated drug identification from pill-level imagery to packaging-level analysis, reflecting real-world pharmacy workflows. Second, it introduces a hybrid feature space that combines physical packaging descriptors and OCR confidence metrics to characterize machine-readability in heterogeneous packaging environments. Third, by embedding these features within a DEA-based evaluation framework, the study provides a structured and reproducible approach for comparative analysis without relying on predictive performance claims.

## 2. Materials and methods

To examine automated antibiotic package identification within real-world pharmacy dispensing workflows, this study employed a structured, multi-stage informatics pipeline. The methodological design follows a sequential logic of physical image capture, feature abstraction, structural grouping, and efficiency evaluation. High-resolution package images were acquired and normalized to reduce acquisition bias. Image-derived physical features and OCR-based textual confidence metrics were then extracted to characterize machine-readability. Unsupervised clustering was applied to explore latent packaging structures without imposing predefined labels, while Data Envelopment Analysis (DEA) was used as an analytical evaluation layer to benchmark the relative efficiency of different feature profiles in supporting correct antibiotic identification. This staged design assigns a distinct and interpretable role to each analytical component within the overall framework.

### 2.1. Imaging Protocol

High-resolution images of commercial antibiotic packages were captured under controlled laboratory conditions (25 ± 1 °C; 55 ± 5% RH) using three device types to reflect diverse operational settings: (1) a full-frame DSLR camera (Nikon D750, 24 MP, Japan) equipped with a 60 mm macro lens, (2) a document camera (Joyusing V500S, 8 MP, China), and (3) a smartphone (Samsung S20, 12 MP, South Korea). All devices were mounted within a custom-built LED lightbox providing uniform, glare-free illumination (950 ± 50 lx), verified using a calibrated lux meter (Uni-Trend UT383, China). White balance was fixed at 6500 K using a 24-patch color chart (Spyder Checkr 24, Datacolor, Germany), and RGB primaries were referenced to CIE standard wavelengths [17,18]. Annotation monitors were color-profiled using a Spyder5 ELITE sensor to maintain display consistency during labeling and inspection [19].

### 2.2. Pre-processing Pipeline

Raw images were archived on a local network-attached storage system (ZyXEL NAS326, China). A four-stage image-processing pipeline was applied to detect and isolate regions of interest (ROI) corresponding to antibiotic packaging:

**Step 1: ROI Detection.** A pretrained YOLOv5 deep-learning model [20] was used to identify and crop rectangular regions containing blister or strip packs. Bounding boxes were resized to 512 × 512 pixels for standardization.

**Step 2: Edge Detection and Thresholding.** Canny edge detection followed by Otsu’s thresholding was applied to generate binary masks emphasizing edge clarity and contrast [21].

**Step 3: Noise Filtering.** Median filtering with a 3 × 3 kernel was used to remove salt-and-pepper noise while preserving OCR-relevant edges.

**Step 4: Image Normalization.** Cropped ROIs underwent histogram stretching for contrast enhancement, yielding high-contrast representations suitable for subsequent text and structural analysis [22].

**Fig 1:**
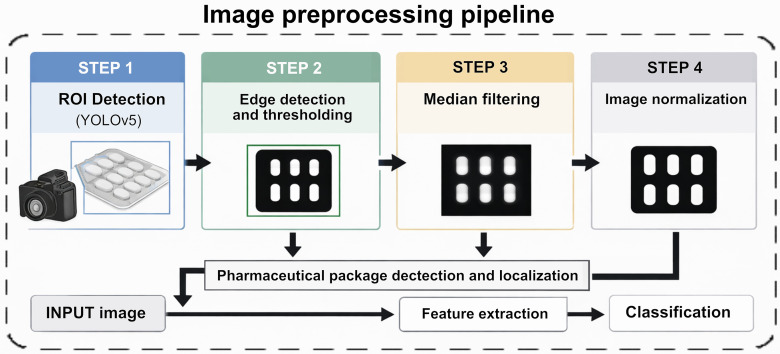
Flowchart illustrating the image pre-processing pipeline for antibiotic packaging analysis, comprising YOLOv5-based detection, edge extraction and thresholding, median noise filtering, and histogram normalization.

Representative visual outputs from each processing stage are shown in Fig 1.

### 2.3. Feature engineering

#### 2.3.1. Packaging area ratio (PAR).

Packaging Area Ratio (PAR) was introduced as a normalization metric to account for variations in package size and image scale across heterogeneous antibiotic products. PAR functions as a design-relevant indicator of spatial consistency, enabling fair comparison of packaging layouts under automated visual inspection. By normalizing the primary packaging area against a standardized reference frame, PAR reduces size-induced bias in both image entropy and OCR-derived features.

Foreground pixels were extracted from binary masks to quantify the primary packaging area (PPA). A fixed-size reference object defined the standard drive area (SDA), corresponding to the pixel area of a credit-card-sized card (85.6 × 53.98 mm) [10]. PAR was computed as:


$$\begin{array}{c} PAR=\frac{PPA}{SDA} \end{array}$$ (1)

where PPA denotes the number of foreground pixels within the segmented packaging region and SDA represents the pixel area equivalent of the standardized imaging frame.

#### 2.3.2. Surface texture and material classification.

The antibiotic packaging dataset comprised two dominant material types with distinct optical and structural properties. Blister packs exhibited smooth surfaces and specular reflections due to rigid transparent polyvinyl chloride (PVC) layers, whereas strip packs showed diffuse, direction-independent reflections arising from opaque laminated aluminum materials. These physical characteristics influence entropy-based classification, as smooth surfaces tend to generate lower entropy values, while diffuse materials produce higher entropy due to random light scatter.

**Fig 2:**
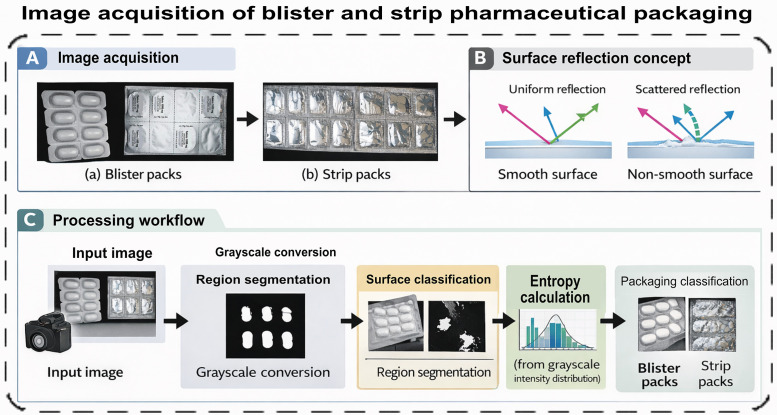
Schematic representation of reflected light behavior and entropy-based classification of pharmaceutical packaging materials.


Images were converted to HSV color space, and the V-channel underwent histogram equalization. Grayscale histograms were generated, and the first three statistical moments (mean, variance, and skewness) were combined with PAR for clustering analysis [11,12]. Small artifacts were removed using a threshold of $T=\text{0}.\text{0}\text{0}\text{5}\times \left(n_{rows}\times n_{cols}\right)$ Image entropy was calculated to quantify surface reflectivity using (Fig 2):

$$\begin{array}{c} H=-\sum p(i){\text{log}}_{\text{2}}p(i) \end{array}$$ (2)

where p(i)represents the normalized frequency of grayscale intensity level i, and Hdenotes the scalar entropy value reflecting texture randomness and surface complexity. Variable definitions supporting reproducibility are summarized in Supplementary Table S1 in S1 File.

#### 2.3.3. Optical character recognition (OCR) features.

Each image was processed using Tesseract OCR v5.3 [13] following adaptive thresholding. The OCR module extracted alphanumeric information from segmented packaging regions, with emphasis on batch codes and product identifiers. The following variables were computed:

- **Character Confidence Score (CCS):** Aggregate OCR confidence assigned at the character level [23].
- **Word Confidence Score (WCS):** Aggregate confidence across contiguous word segments, derived from CCS values [24,25].
- **Levenshtein Edit Distance (LED):** String distance between extracted text and Thai FDA registry entries, with a binary match defined as LED = 0 [26,27].
- **Text-to-PPA Ratio (TPR):** Proportion of OCR-identified text pixels relative to the primary packaging area.

All OCR-derived metrics were included in the composite feature vector for downstream analysis. Definitions, measurement units, and relevance to clustering and DEA modeling are provided in *Supplementary Table S1 in S1 File*, with additional mathematical details summarized in *Supplementary Table S7 in S1 File*.

### 2.4. Study population and sampling

Field surveys conducted in 2021 across four eastern provinces of Thailand identified 36 systemically used antibiotics registered between 2016 and 2020. One brand per active ingredient was selected using simple random sampling. Three units per brand were purchased from community drugstores, yielding a total of 108 physical packages. Full metadata for all sampled products are summarized in *Supplementary Table S2 in S1 File.*

### 2.5. External benchmark and integration

U.S. medicine-use data were obtained from the IQVIA Institute report [28]. Drug classes were mapped to Thai mechanistic groupings, and each physical package was assigned a unique study identifier linking image features, metadata, and market information within a relational database.

### 2.6. Statistical and efficiency analysis

#### 2.6.1. Clustering analysis.

Unsupervised clustering was employed to explore latent structural patterns among antibiotic packages without assuming predefined class labels. K-means clustering was selected for its interpretability, computational efficiency, and suitability for continuous feature spaces derived from image and OCR metrics. All features were normalized using min–max scaling prior to clustering.

The optimal number of clusters was determined using the Calinski–Harabasz (CH) index:


$$\begin{array}{c} CH=\frac{tr\left(B_{k}\right)/(k-\text{1})}{tr\left(W_{k}\right)/(n-k)} \end{array}$$ (3)

where $tr\left(B_{k}\right)\;$and $tr\left(W_{k}\right)$ denote between- and within-cluster dispersion, respectively, k  = number of clusters, and n is the total number of samples [14–16]. The optimal solution (k=9) yielded the highest CH score and was used for subsequent analyses. Cluster characteristics and distributions are reported in *Supplementary Tables S4 in S1 File and S6*.

#### 2.6.2. DEA efficiency modeling.

**Fig 3:**
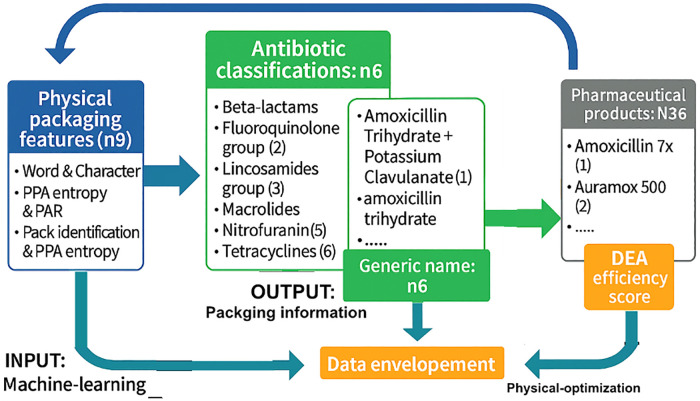
Flowchart illustrating the combined machine-learning and DEA-based analytical framework for packaging evaluation.


DEA was applied as an analytical evaluation layer rather than as a predictive classifier. Each antibiotic package was modeled as a decision-making unit (DMU) transforming multiple physical and OCR-derived inputs into identification-related outputs. DEA analysis employed the CCR model [29], formulated as (Fig 3):

$$\begin{array}{c} {max}_{u,v}\left(\frac{u^{T}y_{\text{0}}}{v^{T}x_{\text{0}}}\right),\;subject\;to\;\frac{u^{T}y_{j}}{v^{T}x_{j}}\leq \text{1},\;\forall_{j};\;u,v\;\geq \text{0} \end{array}$$ (4)

where $y_{j}$and $x_{j}$represent output and input vectors for DMU j, respectively, and uand vare optimized weight vectors [30,31]. DEA efficiency scores (θ) were computed using Microsoft Excel Solver. DMUs with θ=1were classified as efficient, while those with θ<1were considered suboptimal. Full DEA results are provided in Supplementary Table S5 in S1 File.

### 2.7. Methodological Assumptions and Limitations

Several methodological considerations are acknowledged. The dataset reflects commonly used antibiotic products in Thailand and may not capture the full diversity of global packaging designs. Imaging was conducted under controlled laboratory conditions, which may differ from field-based acquisition environments. Additionally, OCR performance may be influenced by language, font variability, and print degradation not fully represented in the current sample. These assumptions provide context for interpretation of subsequent results.

## 3. Results

### 3.1. Clustering of physical and optical features

The optimal number of clusters was determined to be nine, based on the Calinski–Harabasz (CH) index (CH = 1532.6). This configuration offered the best balance of intra-cluster similarity and inter-cluster separation [7].

Cluster 1 was the largest group (n = 9), predominantly composed of blister packs with mid-range entropy (7.2–7.6) and moderate PAR values (1.2–1.45). In contrast, strip packs—which tend to exhibit diffuse reflectivity—were distributed across higher-entropy clusters (H > 7.8), demonstrating higher surface complexity.

**Fig 4:**
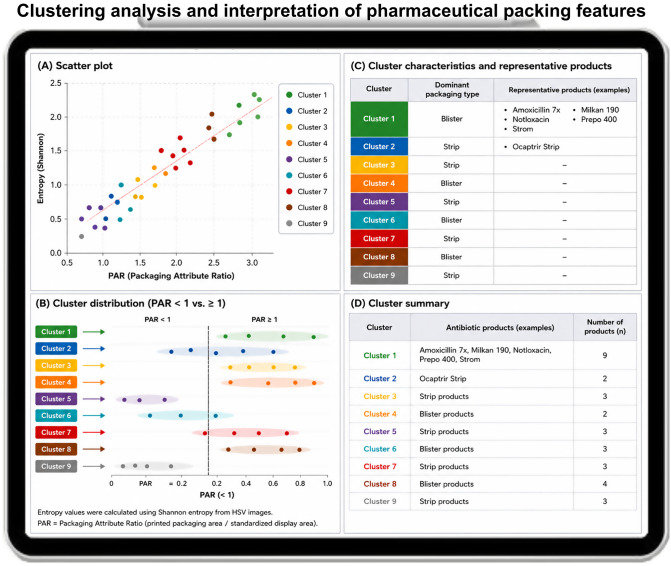
Clustering analysis of pharmaceutical packaging features. (A) Scatter plot of image entropy versus Packaging Area Ratio (PAR) showing the cluster assignments obtained from K-means clustering. Each color represents a different cluster. (B) Distribution of pharmaceutical packaging samples across the nine identified clusters. (C) Summary of the dominant packaging type and the number of products contained within each cluster.

Fig 4A illustrates the strong linear correlation between entropy and PAR across all samples (R² = 0.9072), confirming the underlying clustering mechanism. The left panel displays the entropy–PAR scatter plot used for initial grouping, while the middle panel summarizes the K-means clustering algorithm flow and image inputs. A total of nine clusters were established, each characterized by distinct combinations of PAR and entropy [8,32]. Fig 4B presents the Cluster summary, outlining the composition and statistical features for each group. These patterns align with the material classification established in Section 2.3.2.

- Detailed definitions of feature variables (e.g., entropy, PAR, OCR scores) are provided in Supplementary S1 Table in S1 File.
- Numerical cluster composition and descriptive statistics are summarized in Table 1.
- Interpretation of these findings is further elaborated in Section 4: Discussion.

**Table 1:** Cluster Distribution and Key Statistics of Antibiotic Packages (n = 36).

Cluster	No. of Packs	Dominant Packaging	Entropy (Mean ± SD)	PAR (Mean ± SD)	Notes
**1**	9	Blister	7.34 ± 0.11	1.33 ± 0.07	Smooth, high OCR accuracy
**2**	4	Strip	8.02 ± 0.14	1.12 ± 0.08	Irregular edge reflection
**3**	3	Blister	7.20 ± 0.09	1.47 ± 0.06	Mid-range entropy cluster
**4**	4	Blister	7.60 ± 0.13	1.39 ± 0.05	DEA-efficient cluster
**5**	3	Strip	8.20 ± 0.16	1.28 ± 0.09	High entropy group
**6**	3	Blister	7.41 ± 0.10	1.25 ± 0.07	Uniform OCR region
**7**	3	Strip	8.12 ± 0.12	1.30 ± 0.11	Higher OCR noise
**8**	4	Blister	7.38 ± 0.08	1.36 ± 0.06	DEA-optimal pack type
**9**	3	Strip	8.06 ± 0.17	1.15 ± 0.10	Irregular font/background

All nine clusters identified by the K-means clustering analysis are summarized in Fig 4, including their distribution and dominant packaging characteristics.

The figure presents the clustering results derived from image-based packaging descriptors and provides an overview of cluster composition without additional interpretive commentary.

### 3.2. Evaluation of OCR-derived features for API identification

#### 3.2.1. Confidence metrics in OCR parsing.

Five feature sets—spanning image-based and OCR-derived metrics—were tested for their ability to correctly identify the active pharmaceutical ingredient (API). Among these, Character Confidence Score (CCS) and Word Confidence Score (WCS) achieved the highest predictive accuracy, exceeding 95% in most clusters. Strip-pack-dominated clusters showed lower OCR accuracy, underscoring the influence of surface irregularity [33,34].

A multivariate regression model showed standardized beta coefficients of β = 0.62 (WCS) and β = 0.57 (CCS), indicating strong predictive influence. In contrast, PAR and Text-to-PPA Ratio (TPR) had moderate contributions, while Levenshtein Edit Distance (LED) and text-region density were more significant in noisy OCR settings. These results validate the integration of OCR-based confidence metrics in automated drug package identification pipelines [35,36].

#### 3.2.2 DEA-based efficiency analysis of packaging.

Each antibiotic pack was modeled as a Decision-Making Unit (DMU) within the Data Envelopment Analysis (DEA) framework. Input variables included entropy, PAR, histogram variance, and OCR confidence scores; the output was correct API identification. The efficiency threshold was set at DEA score = 1.00, indicating the most optimized input-output relationship under the model’s assumptions.

Efficiency scores ranged from 0.56 to 1.00, with Clusters 4 and 8 containing the highest number of DEA-efficient DMUs. These clusters also aligned with the most visually coherent and machine-readable packaging types, reinforcing the structural advantages of such designs for digital informatics systems [37,38].

#### 3.2.3. Feature-level predictive power.

To strengthen interpretability, the individual contribution of each feature toward API recognition was evaluated using composite scoring and DEA regression weights. Confidence-based metrics (CCS and WCS) showed dominant influence in predicting correct API identification, consistent across cluster groups. Image-derived inputs such as entropy and PAR contributed indirectly via enhancing readability and structural integrity of the packaging. Cross-referencing these contributions with DEA outputs reveals their weighted impact, confirming the role of composite feature integration.

### 3.3. Packaging type trends and performance patterns

Blister packs demonstrated consistent mid-range entropy and high DEA efficiency, corroborating their smooth and specular surface features described in Section 2.3.2. Conversely, strip packs were associated with higher entropy variation and lower OCR reliability. This dichotomy was evident when plotting entropy versus PAR across pack types (Fig 6A), revealing material–structure interactions that affect image readability and OCR fidelity [36]. Fig 6B adds a feature-wise accuracy analysis, comparing five composite models across all clusters. OCR-based features—specifically WCS and CCS—were the most consistent performers across all cluster types. Additional radar chart data in Fig 7 supports this conclusion.

### 3.4. Feature Contribution Summary

Fig 7 summarizes the impact of each extracted feature—entropy, PAR, CCS, WCS, LED, and TPR—on API identification. Radar plots confirm that WCS and CCS are the dominant predictors, followed by LED. Image-derived metrics (PAR, entropy) contributed indirectly via structural readability and input weighting in the DEA model [38].

## 4. Discussion

### 4.1. Principal findings

This study demonstrates the feasibility of combining physical and optical features—specifically image entropy, Packaging Area Ratio (PAR), and OCR-derived confidence metrics—to cluster antibiotic packaging types and evaluate their relative efficiency for automated identification. Using unsupervised K-means clustering, nine distinct packaging groups were identified, with a strong correlation observed between entropy and PAR (R² = 0.9072), supporting their role as foundational descriptors of packaging structure.

**Fig 5:**
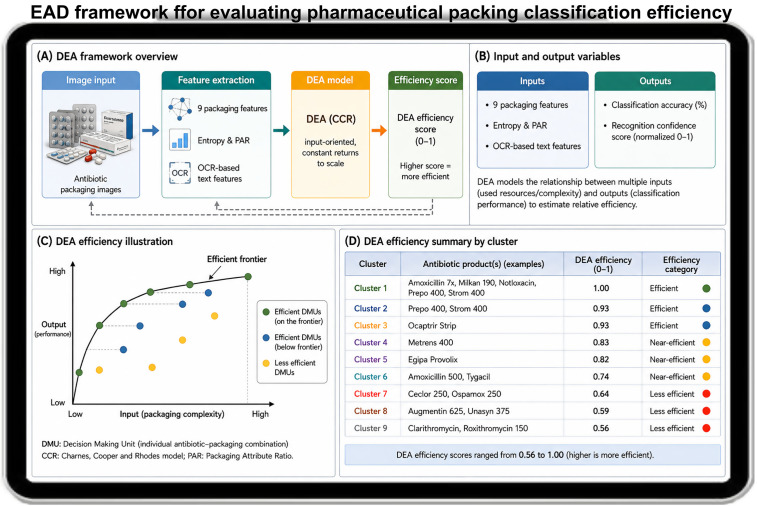
DEA-based evaluation of antibiotic packaging identification efficiency. (A) Relationship between packaging area ratio (PAR), image entropy, and identification-related output signals, illustrating how physical packaging characteristics influence machine-readability. (B) Schematic representation of the DEA framework, where image-derived and OCR-based features are treated as inputs and identification performance as output, positioning DEA as an evaluation layer rather than a classifier. (C) Summary of DEA efficiency scores across packaging clusters under comparable analytical conditions. The figure illustrates how DEA can be used to benchmark packaging configurations based on image-derived and OCR-based characteristics.

DEA modeling further identified Clusters 4 and 8 as DEA-efficient, indicating favorable input–output alignment under comparable analytical conditions. OCR-based metrics, including Word Confidence Score (WCS) and Character Confidence Score (CCS), showed stronger associations with correct API identification than image-derived features alone, highlighting the value of text-based confidence information when integrated into a composite informatics framework. Fig 5 reinforces these findings by illustrating the role of DEA as an evaluation layer linking image- and OCR-derived inputs to identification outcomes, while Table 1 provides cluster-level statistical validation. Additional details on cluster composition, feature distributions, and packaging–API traceability are provided in *Supplementary Tables S4–S6 in S1 File*.

### 4.2. Novelty and Technical Contribution

This work advances existing literature in several important ways. First, it introduces a hybrid feature space that integrates entropy-based texture measures, geometric area descriptors (PAR), and OCR confidence metrics, specifically tailored to the analysis of pharmaceutical packaging rather than pill-level imagery. Second, by embedding these features within a DEA-based efficiency framework, the study bridges computational image analysis with decision science, enabling structured benchmarking of packaging configurations without reliance on supervised classification models.

**Fig 6:**
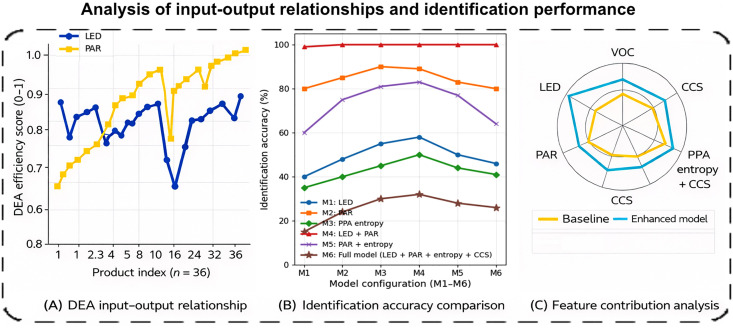
Comparative variation of feature–output relationships across packaging configurations. (A) Relative variation of DEA-related input and output measures across pharmaceutical products, illustrating sensitivity of feature configurations rather than performance evaluation. (B) Comparative trends of identification consistency across cluster groups and feature combinations. The figure is intended to illustrate behavioral variation and relative sensitivity of different feature sets, not to provide accuracy-based performance benchmarking.

**Fig 7:**
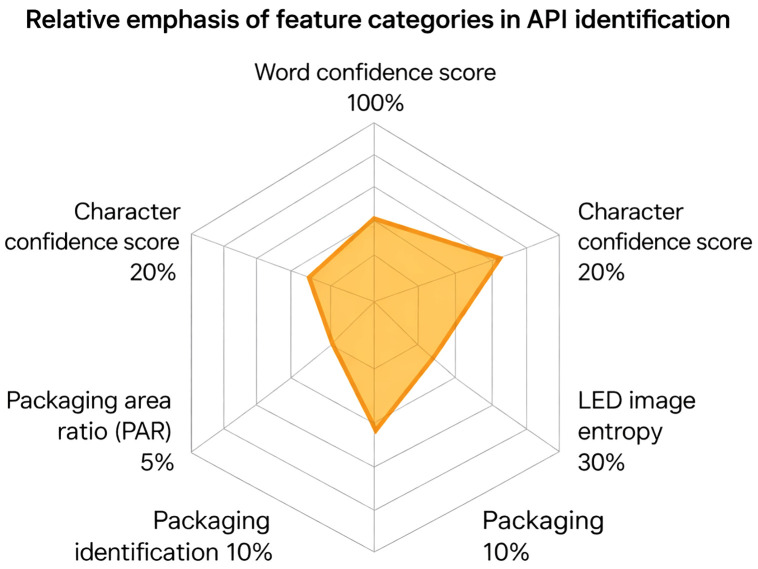
Relative emphasis of feature categories in API identification. Radar chart summarizing the relative emphasis of different feature categories (OCR confidence scores, entropy-based image features, packaging area ratio, and PPA-derived measurements) used within the proposed framework. The percentages represent descriptive, normalized summaries intended to illustrate relative feature emphasis rather than statistically learned importance or performance-based weighting.

To our knowledge, this study is among the early works to provide a comprehensive feature-to-efficiency mapping (Fig 6) and a cross-validated composite feature emphasis summary (Fig 7) within a unified analytical framework. Compared with prior studies that primarily rely on shape- or color-based clustering [7,8], the proposed multi-feature integration supports broader generalisability and improved automation potential. *Supplementary Table S7 in S1 File* further clarifies the mathematical logic underlying the DEA variable construction, facilitating replication and adaptation of the framework to other packaging or labeling contexts.

### 4.3. Clinical and Informatics Implications

From a clinical informatics perspective, the findings highlight how packaging structure—particularly surface smoothness, reflectivity, and text clarity—can influence OCR reliability and, by extension, automated medication identification. Packaging clusters associated with higher DEA efficiency and stronger OCR confidence metrics may inform future considerations for machine-readable packaging design in digital dispensing environments. In community pharmacy or field-based settings where optical capture is commonly used, such insights may help improve robustness against variability in image acquisition and packaging heterogeneity.

These implications should be interpreted as analytical insights intended to inform packaging evaluation and design, rather than as direct clinical or regulatory decision-making recommendations. Nevertheless, the observed associations align with broader international efforts, including WHO and GS1 initiatives, aimed at improving digital readability and interoperability of pharmaceutical labeling [36,37]. Cross-cluster comparisons and DEA unit-level analyses supporting these interpretations are detailed in *Supplementary Tables S5 and S6 in S1 File*.

### 4.4. Limitations and Future Directions

Several limitations should be acknowledged. The sample size (n = 36) may not fully capture the diversity of antibiotic packaging encountered across global supply chains. While the analysis prioritized packaging-level physical and optical features, content-level variability—such as font styles, multilingual labeling, and foil texture heterogeneity—was not exhaustively examined. Future studies should expand the dataset to include a broader range of international packaging formats and explore the integration of deep learning approaches for automated segmentation and feature extraction.

Although DEA proved effective for efficiency evaluation in this context, future work may incorporate multi-objective optimization frameworks to balance digital readability with additional considerations such as packaging cost, environmental impact, and manufacturing constraints. Ensemble clustering techniques could also be explored to assess the stability of packaging groupings across datasets. Detailed methodological descriptions provided in the Supplementary Materials are intended to support future comparative benchmarking and extension of the proposed framework.

### 4.5. Reproducibility and Methodological Transparency for Implementation

The proposed framework emphasizes reproducibility, transparency, and interpretability to facilitate adoption and extension by other researchers. All variables, equations, and analytical parameters are fully defined in Section 2 and linked to corresponding Supplementary Tables. Figs 4–7 were designed to clarify feature derivation, clustering logic, and efficiency evaluation, while Table 1 summarizes cluster characteristics in a reader-accessible format without altering analytical outcomes.

The radar chart in Fig 7 provides a descriptive summary of relative feature emphasis within the framework and may support exploratory decision-making in packaging redesign contexts. Together with Supplementary Tables S1–S8 in S1 File and the detailed methodological descriptions provided in the Supplementary Appendix, these elements enable independent understanding and replication of the analytical workflow.

## 5. Conclusion

This study presents a composite informatics framework that integrates image-derived features and OCR-based confidence metrics to examine the packaging characteristics of 36 commonly used antibiotic products. The analysis indicates that image entropy and Packaging Area Ratio (PAR) are informative descriptors for exploratory clustering of packaging types, while OCR-based measures, including Word Confidence Score (WCS) and Character Confidence Score (CCS), are strongly associated with correct API identification within the proposed analytical framework. Using a combination of K-means clustering and Data Envelopment Analysis (DEA), nine distinct packaging clusters were identified, with Clusters 4 and 8 exhibiting comparatively favorable input–output efficiency profiles under the study’s assumptions [7,34,35].

By integrating physical packaging descriptors and OCR confidence metrics within a DEA-based evaluation layer, the proposed framework offers a replicable and mathematically grounded approach for assessing the digital readability of pharmaceutical packaging. The combined use of visual and textual feature sets supports more robust analytical characterization in environments where heterogeneous packaging designs complicate automated identification. Detailed methodological descriptions, supplementary analyses, and cluster-level summaries provided in Supplementary Tables S1–S8 in S1 File are intended to support transparency and independent replication of the analytical workflow [6,33,37,38].

Overall, the findings suggest that improved alignment between packaging design and machine-readable characteristics may enhance the reliability of digital drug identification systems. As an analytical and benchmarking tool, the proposed framework provides a structured basis for exploring packaging-level factors relevant to medication safety, digital health informatics, and pharmaceutical supply chain analysis, with potential applicability to broader contexts where visual and textual information must be jointly evaluated [39,27,8,36–38].

## Supporting information

S1 File Supplementary Methods, Supplementary Tables S1–S8, and Supplementary Note. This file contains supplementary methods for image acquisition and preprocessing, feature extraction procedures, OCR metrics, DEA configuration, Supplementary Tables S1–S8, and supplementary interpretation of DEA results.
(DOCX)

S2 Dataset Minimal dataset underlying the findings of the study. This dataset contains raw image-derived variables, OCR-derived variables, clustering assignments, DEA input/output variables, and the values used to generate the reported figures and descriptive statistics reported in the manuscript.
(ZIP)
