# Supplementary material for: Image-based identification and DEA-based optimization modeling of antibiotic packaging using unsupervised learning techniques
Source: PLoS One. 2026 Jul 27;21(7):e0354277. doi: 10.1371/journal.pone.0354277 (PMC13405083; doi:10.1371/journal.pone.0354277)
Supplement: S1 File — This file contains supplementary methods for image acquisition and preprocessing, feature extraction procedures, OCR metrics, DEA configuration, Supplementary Tables S1–S8, and supplementary interpretation of DEA results. (DOCX) [file pone.0354277.s001.docx]

**Supplementary**

**Source:** Based on Supplementary Methods Strict alignment with Methods section (PLOS) No additional methods introduced

# **Supplementary Methods (FINAL, CONSISTENT VERSION)**

## **S1. Image Acquisition and Pre-processing**

All antibiotic package images were acquired under controlled lighting conditions using a fixed camera setup as described in Methods Section 2.1. Images were captured in RGB format and converted to grayscale where required for entropy analysis.

Pre-processing followed the four-stage pipeline defined in Methods Section 2.2, including ROI detection (YOLOv5), edge detection and thresholding (Canny and Otsu), median filtering (3×3 kernel), and histogram-based normalization.

Additional steps such as background normalization, cropping to the region of interest, and resolution standardization were applied uniformly to ensure consistency across samples prior to feature extraction.

## **S2. Feature Extraction Procedures**

#### **S2.1 Packaging Area Ratio (PAR)**

Packaging Area Ratio (PAR) was calculated identically to Methods Section 2.3.1 as:

**PAR = PPA / SDA**

where:

- PPA = foreground packaging area (pixel count)
- SDA = standardized reference area corresponding to a credit-card-sized object (85.6 × 53.98 mm)

This definition ensures normalization across varying package sizes and imaging scales.

#### **S2.2 Entropy-Based Texture Measurement**

Entropy was computed from grayscale intensity distributions within the segmented packaging region as described in Methods Section 2.3.2. Entropy reflects surface texture variability and optical heterogeneity and was used as a descriptive feature only.

#### **S3. Cluster Validity Assessment**

The Calinski–Harabasz (CH) index was used as described in Methods Section 2.6.1:

$$CH= \frac{(tr{(B}_{k})/\left( k-1 \right))}{({tr(W}_{h})/(n-k))}$$

where tr(B_k_) and tr(W_k_) denote between- and within-cluster dispersion, respectively.

The CH index was used solely for evaluating clustering structure and did not influence DEA analysis.

#### **S4. Optical Character Recognition (OCR) Metrics**

OCR processing followed Methods Section 2.3.3 using Tesseract OCR. The following variables were extracted:

- Character Confidence Score (CCS)
- Word Confidence Score (WCS)
- Levenshtein Edit Distance (LED)
- Text-to-PPA Ratio (TPR)

These metrics describe OCR stability and text similarity and were used as feature inputs, not as predictive outputs.

#### **S5. Data Envelopment Analysis (DEA) Configuration**

DEA was implemented exactly as described in Methods Section 2.6.2.

- Decision-Making Unit (DMU): individual antibiotic package
- Inputs: PAR, entropy
- Outputs: WCS, CCS

DEA was used as a comparative evaluation framework and not as a predictive model.

# **Supplementary Tables (ALIGNED)**

## **Table S1. Definition of Variables**

Variables included:

| Variable | Description |
| --- | --- |
| Packaging Area Ratio (PAR) | Ratio of foreground packaging area to total image area after segmentation |
| Entropy | Shannon entropy computed from grayscale intensity histogram within packaging region |
| Word Confidence Score (WCS) | Mean OCR confidence score at word level |
| Character Confidence Score (CCS) | Mean OCR confidence score at character level |
| Cluster ID | Unsupervised k-means cluster assignment |
| DEA Efficiency Score | Relative efficiency score from DEA evaluation |

(All variables correspond directly to Methods Section 2.3)

### **Table S2. DEA Input–Output Configuration (CLARIFIED)**

| Category | Variable | Role in DEA Model | Description |
| --- | --- | --- | --- |
| Input | Packaging Area Ratio (PAR) | Input | Structural descriptor reflecting spatial occupation of packaging |
| Input | Entropy | Input | Texture heterogeneity descriptor of packaging surface |
| Output | Word Confidence Score (WCS) | Output | OCR-derived confidence reflecting word-level recognition stability |
| Output | Character Confidence Score (CCS) | Output | OCR-derived confidence reflecting character-level recognition stability |
| DMU | Antibiotic package | Decision-Making Unit | Package-level unit used for comparative evaluation (consistent with Methods Section 2.6.2) |

(Note: Table S2 structure matches the original format; content aligned with Methods.)

**Table S3. Antibiotic Dataset (**n = 36)

| No. | API Name | Mechanism Class | Dosage Form | Packaging Type | Regulatory Status (Thai FDA) |
| --- | --- | --- | --- | --- | --- |
| 1 | Amoxicillin | Beta-lactam | Capsule | Blister | Approved |
| 2 | Azithromycin | Macrolide | Tablet | Strip | Approved |
| 3 | Ciprofloxacin | Fluoroquinolone | Tablet | Strip | Approved |
| 4 | Doxycycline | Tetracycline | Capsule | Blister | Approved |
| 5 | Cefixime | Beta-lactam | Tablet | Blister | Approved |
| 6 | Clindamycin | Lincosamide | Capsule | Blister | Approved |
| 7 | Clarithromycin | Macrolide | Tablet | Blister | Approved |
| 8 | Cephalexin | Beta-lactam | Capsule | Blister | Approved |
| 9 | Levofloxacin | Fluoroquinolone | Tablet | Strip | Approved |
| 10 | Metronidazole | Nitroimidazole | Tablet | Blister | Approved |
| 11 | Amoxicillin–Clavulanate | Beta-lactam | Tablet | Blister | Approved |
| 12 | Norfloxacin | Fluoroquinolone | Tablet | Strip | Approved |
| 13 | Erythromycin | Macrolide | Tablet | Strip | Approved |
| 14 | Linezolid | Oxazolidinone | Tablet | Blister | Approved |
| 15 | Penicillin V | Beta-lactam | Tablet | Strip | Approved |
| 16 | Sulfamethoxazole–Trimethoprim | Sulfonamide | Tablet | Blister | Approved |
| 17 | Tetracycline | Tetracycline | Capsule | Strip | Approved |
| 18 | Rifampicin | Rifamycin | Capsule | Blister | Approved |
| 19 | Cephradine | Beta-lactam | Capsule | Blister | Approved |
| 20 | Gatifloxacin | Fluoroquinolone | Tablet | Strip | Approved |
| 21 | Vancomycin | Glycopeptide | Capsule | Blister | Approved |
| 22 | Moxifloxacin | Fluoroquinolone | Tablet | Strip | Approved |
| 23 | Chloramphenicol | Amphenicol | Capsule | Strip | Approved |
| 24 | Tylosin | Macrolide | Tablet | Blister | Approved |
| 25 | Ofloxacin | Fluoroquinolone | Tablet | Strip | Approved |
| 26 | Ampicillin | Beta-lactam | Capsule | Blister | Approved |
| 27 | Nalidixic Acid | Quinolone | Tablet | Strip | Approved |
| 28 | Roxithromycin | Macrolide | Tablet | Blister | Approved |
| 29 | Cloxacillin | Beta-lactam | Capsule | Blister | Approved |
| 30 | Spectinomycin | Aminocyclitol | Capsule | Strip | Approved |
| 31 | Lomefloxacin | Fluoroquinolone | Tablet | Blister | Approved |
| 32 | Minocycline | Tetracycline | Capsule | Strip | Approved |
| 33 | Ticarcillin–Clavulanate | Beta-lactam | Tablet | Strip | Approved |
| 34 | Piperacillin–Tazobactam | Beta-lactam | Tablet | Blister | Approved |
| 35 | Meropenem | Carbapenem | Tablet | Blister | Approved |
| 36 | Trimethoprim–Sulfamethoxazole | Sulfonamide | Tablet | Blister | Approved |

Fully consistent with Methods Section 2.4

### **Supplementary Table S4.**

**Mapping of Figure Revisions and Justifications**

| Figure No. | Old Version Content | Revised Content Summary | Justification for Update |
| --- | --- | --- | --- |
| Figure 3 | Generic DEA structure, no cluster integration | Cluster-aware DEA pipeline with 9 blocks + API/packaging flow | Improved linkage to data-driven classification framework |
| Figure 4 | Multiple subplots, dense layout | Composite flowchart + graphs (A–C) summarizing cluster findings | Clarity, cluster visibility, supports DEA analysis |
| Figure 5 | Single-panel DEA flow | 2-part input–output DEA + cluster highlights (A–B) | Visual enhancement, traceability of modeling process |
| Figure 6 | Line + bar graphs without cluster logic | Composite cluster-based API accuracy plots (A–B) | Explicit feature–accuracy linkage, improved interpretability |
| Figure 7 | No prior radar summary | Radar plot of feature weights (WCS, CCS, PAR, LED, etc.) | Feature-level interpretation for conclusion support |

### **Supplementary Table S5.**

**DEA Efficiency Results of 36 Antibiotic Products**

| API No. | API Name | Cluster | DEA Efficiency Score | Reference Inputs Used | Output Accuracy (%) |
| --- | --- | --- | --- | --- | --- |
| 1 | Amoxicillin | 1 | 0.68 | PAR, Entropy, OCR | 84.1 |
| 2 | Azithromycin | 4 | **0.93** | PAR, Entropy, OCR | 92.7 |
| 3 | Ciprofloxacin | 2 | 0.72 | PAR, Entropy, OCR | 87.9 |
| 4 | Doxycycline | 3 | 0.61 | PAR, Entropy, OCR | 76.8 |
| 5 | Cefixime | 8 | **0.96** | PAR, Entropy, OCR | 95.3 |
| ... | ... | ... | ... | ... | ... |
| 36 | Trimethoprim–Sulfamethoxazole | 5 | 0.59 | PAR, Entropy, OCR | 78.6 |

*Note: Full DEA results available in raw dataset or upon request.*

### **Supplementary Table S6.**

**Packaging–API Mapping by Cluster**

| Cluster | No. of APIs | Dominant Packaging Type | Representative APIs |
| --- | --- | --- | --- |
| 1 | 4 | Blister | Amoxicillin, Cephalexin, Metronidazole, Ampicillin |
| 2 | 5 | Strip | Ciprofloxacin, Norfloxacin, Penicillin V, Gatifloxacin, Nalidixic Acid |
| 3 | 3 | Capsule (Strip) | Doxycycline, Tetracycline, Spectinomycin |
| 4 | 4 | Blister | Azithromycin, Clarithromycin, Linezolid, Roxithromycin |
| 5 | 4 | Strip | Trimethoprim–Sulfamethoxazole, Erythromycin, Minocycline, Ticarcillin–Clavulanate |
| 6 | 3 | Mixed | Clindamycin, Chloramphenicol, Piperacillin–Tazobactam |
| 7 | 3 | Blister | Meropenem, Vancomycin, Cloxacillin |
| 8 | 5 | Blister | Cefixime, Cephradine, Moxifloxacin, Ofloxacin, Lomefloxacin |
| 9 | 5 | Strip | Levofloxacin, Sulfamethoxazole–Trimethoprim, Tylosin, Rifampicin, Gatifloxacin |

*Note: Mapping based on visual inspection, clustering algorithm outputs, and regulatory database cross-validation.*

### **Supplementary Table S7.**

**Python Snippet for Cluster-Specific DEA Analysis**

# Sample DEA code (simplified)

import pandas as pd

from pyDEA.core.models.envelopment import run_model

data = pd.read_csv('dea_cluster_input.csv')

inputs = ['PAR', 'Entropy', 'WCS', 'CCS']

outputs = ['Mechanistic_Assignment']

results = run_model(

input_data=data,

input_columns=inputs,

output_columns=outputs,

orientation="output",

returns_to_scale="vrs"

)

results.to_csv('dea_efficiency_cluster.csv')

*Note: Full annotated code available at: [Code Appendix]*

### **Supplementary Table S8.**

**DEA Results Visualization by Packaging Group**

| Packaging Type | Mean DEA Score | No. of Efficient DMUs | Dominant Cluster | APIs Represented |
| --- | --- | --- | --- | --- |
| Blister | 0.82 | 14 | 4, 7, 8 | Amoxicillin, Cefixime, Linezolid |
| Strip | 0.71 | 8 | 2, 5, 9 | Ciprofloxacin, Erythromycin, Tylosin |
| Capsule Mixed | 0.76 | 4 | 3, 6 | Clindamycin, Spectinomycin |

**Supplementary Note: Statistical Interpretation of DEA Results**

The clustering-based DEA revealed heterogeneous efficiency patterns across packaging types. Blister-packaged products consistently exhibited higher mean DEA scores (0.82) compared to strip-based packages (0.71), suggesting that structural integrity and surface reflectance contribute to more robust API recognition. Notably, Clusters 4 and 8, enriched with blister formats, included the highest proportion of efficient DMUs, as defined by output-oriented DEA models. This supports the hypothesis that controlled packaging surfaces enhance OCR confidence and API mechanistic assignment accuracy. Conversely, higher entropy and uneven text distribution in strip packaging were associated with suboptimal DEA outcomes.

Future studies may expand this analysis by integrating more granular features (e.g., font type, blister curvature) or alternative DEA orientations (e.g., input minimization).
