# Supplementary material for: Image-based identification and DEA-based optimization modeling of antibiotic packaging using unsupervised learning techniques
Source: PLoS One. 2026 Jul 27;21(7):e0354277. doi: 10.1371/journal.pone.0354277 (PMC13405083; doi:10.1371/journal.pone.0354277)
Supplement: Attachment — Submitted filename: Response to Reviewers.pdf [file pone.0354277.s004.pdf]

Reviewer's Comment-to-Revision Mapping Table (Full Document Coverage)

Reviewer Comment / Concern	Location in Manuscript	Revision Implemented	Reviewer
**1. Insufficient explanation of the rationale and background behind API identification study.**	Abstract & Introduction	Background revised in Abstract and Introduction to emphasize relevance of API identification to medication safety and AMR in LMIC settings.	Reviewer #2
**2. Missing technical descriptions for image processing workflow.**	Methods Section 2.3	A detailed workflow (binary imaging, PAR, entropy, OCR pipeline) added in Methods. Figures 2–4 visually support each component.	Reviewer #1
**3. Lack of explanation on the K-means clustering output and its interpretation.**	Results Section 3.1	Descriptive narrative added in 3.1 on entropy-PAR clusters and relevance to packaging standardization.	Reviewer #3
**4. Need clearer explanation of what entropy and PAR signify in real-world packaging.**	Discussion Section	Discussion updated with interpretation of entropy (surface complexity) and PAR (size standardization) for practical application.	Reviewer #3
**5. DEA application was novel but underdeveloped in the manuscript.**	Results Section 3.2.2 + Discussion	Section 3.2.2 expanded to explain DEA inputs/outputs, Figure 5 redesigned and captioned. Discussion now connects DEA to package traceability strategy.	Reviewer #1
**6. Abstract too long and lacking clarity in objectives and outcomes.**	Abstract	Abstract rewritten in ≤250 words format. Structured into Background–Objective–Methods–Results–Conclusion.	Reviewer #2
**7. Discussion lacks forward-looking implication on AI-**	Discussion (final paragraph)	Closing paragraph added linking findings to AI-assisted verification, robotic pharmacy, and reduced medication error.	Reviewer #2

**pharmacy integration.**			
**8. Reference list not up to date with 2023–2025 papers.**	All sections	Updated references [8], [9], and [41–43] to include recent literature from 2021–2024 on OCR, AI verification, and Thai antimicrobial stewardship.	Reviewer #1
**9. Figures lacked captions detailing what insight they provide.**	Figures 4–7	Captions updated to include interpretive summaries (e.g., "entropy range", "DEA frontier", "feature contribution", etc.).	Reviewer #3
**10. Insufficient description of Thailand-specific pharmaceutical context.**	Introduction	Added text explaining proliferation of generics and label inconsistencies in Thai community pharmacy setting.	Reviewer #2
**11. Reviewer requested comparison to other visual drug ID approaches.**	Discussion	Brief comparative mention of pill-based image recognition studies [4–6] added to frame packaging-level novelty.	Reviewer #1
**12. No mention of study limitations or error sources.**	Discussion (final section)	Limitation paragraph added: image quality variability, OCR misread potential, dataset limited to antibiotics.	Reviewer #3
**13. DEA equation requires full variable definitions and reproducibility clarity.**	Methods Section 2.6.2 + Figure 3	Equation (4) clarified with detailed explanation of all variables (x, y, u, v, E_0), modeling rationale, and Solver implementation. Figure 3 redesigned to reflect DEA logic and 9-cluster input-output flow.	Reviewer #1
**14. Incomplete coverage of all Methods subsections.**	Methods Section (2.1–2.6)	All subsections restored, restructured in sequence (2.1–2.6), including new definitions for variables, imaging setup, and dual packaging types.	Reviewer #3

**15. Reviewer mentioned unclear variable use in equations and lack of mathematical clarity.**	Methods Sections 2.5–2.6 (Entropy, DEA)	Definitions of H, p(i), x_i, y_r, u_r, v_i added inline; structured explanations provided in paragraph form for reproducibility and clarity.	Reviewer #1
**16. Figures for Results section lack clarity and interpretability.**	Results Figures 4–7	All key results figures redesigned using composite style: (A) modeling logic or scatter analysis and (B) summary/metrics. Improved color, legend, and text clarity.	Reviewer #2
**17. Clarify contribution of features to API recognition outcomes.**	Results Section 3.2.3 + Figure 6–7	Feature impact summarized via radar plot (Figure 7), supported by line accuracy trends across clusters (Figure 6B).	Reviewer #2
**18. Figures should show relationship between machine-learning and physical packaging data.**	Results Section 3.2.1 + Figure 6A	Figure 6A visualizes DEA input–output divergence between ML-derived and packaging-derived values for 36 products.	Reviewer #1
**19. Reviewer requested variable summary or visual glossary.**	Supplementary Table S1	A new Supplementary Table S1 added summarizing all variables, definitions, units, and measurement origins for reproducibility and clarity.	Reviewer #1
**20. Unclear cross-linking between Methods and Figures in Results**	Figure Legends + Methods 2.3–2.6	Cross-references inserted (e.g., “see Figure 4B”) in Methods, plus in-text figure calling added in Results.	Reviewer #3
**21. Feature–API predictive logic missing in Results**	Results Section 3.2.3	Added new sub-section 3.2.3 titled “Feature-Level Predictive Power” to summarize contributions of individual features to API prediction.	Reviewer #2
**22. Concern on clarity of composite figures vs original graphics**	Results Figures 4–7	Composite figures (e.g., 4, 5, 6) designed to preserve original analysis logic; older plots referenced in legend to show derivation.	Reviewer #1

**23. Clustering section lacks numerical summary or descriptive tabulation.**	Results Section 3.1	**Table 1** was newly created to enhance interpretability of clustering results, based on metrics already presented in the original manuscript. It summarizes entropy and PAR statistics per cluster and supports Figure 4 interpretation.	Reviewer #3
