# Supplementary material for: Image-based identification and DEA-based optimization modeling of antibiotic packaging using unsupervised learning techniques
Source: PLoS One. 2026 Jul 27;21(7):e0354277. doi: 10.1371/journal.pone.0354277 (PMC13405083; doi:10.1371/journal.pone.0354277)
Supplement: Attachment — Submitted filename: Rebuttal_Letter_221025.pdf [file pone.0354277.s005.pdf]

Rebuttal Letter to the Academic Editor and Reviewers

Manuscript ID: PONE-D-25-36293R1

Title: Image-Based Identification and DEA-Based Optimization Modeling of Antibiotic Packaging Using Unsupervised Learning Techniques

Cover Note

Dear Dr. Renier Mendoza and Esteemed Reviewers,

We sincerely thank the Academic Editor and the Reviewers for their thoughtful and constructive feedback on our manuscript. We have carefully and comprehensively revised the paper to address every comment in detail. The revised version has been substantially improved in clarity, methodological rigor, and contextual interpretation.

We believe that these revisions fully address all reviewer concerns and bring the manuscript in line with PLOS ONE's publication standards.

Thank you very much for your time, consideration, and valuable input.

Sincerely,

Ms. Suthabordee Muongmee
Corresponding Author
Faculty of Pharmaceutical Sciences, Burapha University
Email: suthabordee@go.buu.ac.th

Reviewer-Specific Responses

Reviewer #1

Reviewer Comment	Author Response	Location in Revised Manuscript
Missing technical descriptions for image-processing workflow.	A detailed workflow was added in Methods Section 2.3, describing binary image conversion, PAR and entropy calculations, and OCR integration. New Figures 2–4 visually illustrate each component.	Methods 2.3; Figures 2–4

DEA section underdeveloped and lacking clarity.	Section 3.2.2 was expanded to explain DEA inputs/outputs, the efficiency frontier, and Solver implementation. Figure 5 redesigned. Discussion links DEA to packaging traceability.	Results 3.2.2; Figure 5; Discussion
DEA equation lacks variable definitions and reproducibility.	Equation (4) revised with definitions of variables (x, y, u, v, E_0). Figure 3 redesigned accordingly.	Methods 2.6.2; Figure 3
Need visual summary of variables.	Added Supplementary Table S1 summarizing all variable definitions, units, and origins.	Supplementary Table S1
Figures should relate ML data to physical packaging.	New Figure 6A visualizes DEA input–output divergence between ML-derived and packaging-derived values for 36 antibiotic products.	Results 3.2.1; Figure 6A

Reviewer #2

Reviewer Comment	Author Response	Location in Revised Manuscript
Insufficient background explaining rationale for API identification.	Abstract and Introduction revised to emphasize relevance to medication safety and AMR in LMICs.	Abstract; Introduction
Abstract too long and unclear.	Abstract rewritten (≤250 words) with structured format: Background–Objective–Methods–Results–Conclusion.	Abstract
Discussion lacks forward-	Added closing paragraph	Discussion (final

looking implication for AI-pharmacy integration.	linking to AI-assisted verification, robotic pharmacy systems, and reduction of medication errors.	paragraph)
Results figures unclear or lacking interpretability.	Results Figures 4–7 redesigned with improved labeling, legends, and color contrast for interpretability.	Figures 4–7
Clarify contribution of features to API recognition outcomes.	Added new subsection 3.2.3 “Feature-Level Predictive Power” with radar and line plots (Figures 6B–7).	Results 3.2.3; Figures 6B–7

Reviewer #3

Reviewer Comment	Author Response	Location in Revised Manuscript
K-means clustering output lacks interpretation.	Section 3.1 revised with narrative explanation of entropy–PAR clusters and relevance to packaging standardization.	Results 3.1
Explain meaning of entropy and PAR in real-world packaging.	Discussion expanded to interpret entropy as surface complexity and PAR as physical size standardization.	Discussion
Discussion missing study limitations.	New Limitations paragraph added at end of Discussion addressing dataset scope, OCR misreads, and image variability.	Discussion (final section)
Clustering lacks tabulated summary.	Added Table 1 summarizing entropy and PAR statistics per cluster	Results 3.1; Table 1

	for interpretability of Figure 4.	
Incomplete coverage of Methods subsections.	Methods 2.1–2.6 restructured sequentially with clarified definitions, imaging setup, and dual packaging descriptions.	Methods 2.1–2.6

Reviewer's Comment-to-Revision Mapping Table (Full Document Coverage)

This table consolidates all reviewer concerns, manuscript locations, and implemented revisions for full transparency, as previously provided in the Response to Reviewers file.

1. Background revised in Abstract & Introduction emphasizing API identification and AMR relevance (Reviewer #2)
2. Image-processing workflow detailed in Methods 2.3 with Figures 2–4 (Reviewer #1)
3. Clustering interpretation added in Results 3.1 (Reviewer #3)
4. DEA expanded and clarified in 3.2.2 with new Figure 5 (Reviewer #1)
5. Abstract rewritten (≤250 words) with structured format (Reviewer #2)
6. Discussion expanded to cover AI-pharmacy implications and study limitations (Reviewers #2, #3)
7. References updated (2021–2024) and figure captions improved (Reviewer #1, #3)
8. Supplementary Table S1 summarizing all variables added (Reviewer #1)
9. Table 1 summarizing cluster metrics added (Reviewer #3)
10. Methods reorganized (2.1–2.6) and cross-linking to Figures added (Reviewer #3)

Summary Statement

All reviewer suggestions have been incorporated. The manuscript was extensively revised in structure, figures, and updated references (2021–2024). We are grateful for the reviewers' insightful feedback, which has substantially strengthened the methodological depth and contextual impact of the study.
