# Supplementary material for: Image-based identification and DEA-based optimization modeling of antibiotic packaging using unsupervised learning techniques
Source: PLoS One. 2026 Jul 27;21(7):e0354277. doi: 10.1371/journal.pone.0354277 (PMC13405083; doi:10.1371/journal.pone.0354277)
Supplement: Attachment — Submitted filename: PLOS ONE review_.pdf [file pone.0354277.s007.pdf]

The manuscript has improved substantially in terms of writing quality; however, it appears that the revisions were made primarily to satisfy reviewer comments rather than to enhance overall readability for the intended audience. The authors seem to assume a high level of prior familiarity with the topic, which is inconsistent with their claim that the work is novel and represents a first-time application. Several passages read as responses to reviewer critiques and would be more appropriately placed in the rebuttal letter rather than in the main manuscript.

Overall, the study is interesting, and the results offer valuable insights into the future of automated dispensing systems. However, the presentation of the results is often confusing and contains numerous inconsistencies and errors. My specific comments and suggestions are listed below:

1. The DOI in Reference #2 is incorrect.
2. Citations in the Introduction should be improved. When making specific claims, the corresponding references should be clearly identified rather than grouped together in a single citation.
3. The discussion of DEA in the Introduction remains insufficient. The authors should explain how DEA enables comparative efficiency assessment without assuming predefined functional relationships, and clarify what is meant by such predefined relationships.
4. Provide a brief description of the image-based metrics in the Introduction.
5. Several terms in Section 2.1 are not defined.
6. The term *"steps"* is used in the image, whereas *"stages"* is used in the caption.
7. The term *"mipelee"* in Figure 1 appears to be a typographical error. If not, its meaning should be explained.
8. The flowchart shown below the STEPS in Figure 1 should be explained in the caption.
9. Insert a brief description of Section 2.3 before introducing its subsections.
10. HSV is not defined when first introduced.
11. In Equation (2), clarify the index over which the summation is taken.
12. Figure 2 contains typographical errors: *"incicient," "smooh,"* and *"greeyscale."*
13. In Figure 2C, clarify what is meant by *"locate"* an algorithm.
14. The captions above the images in Figure 2C are too small to read. Additionally, in the final image, it is unclear which regions correspond to smooth and not smooth areas. The images are overly simplified, and the captions lack sufficient descriptive detail.
15. Provide an explanation of adaptive thresholding in Section 2.3.3.
16. The definition of API appears in Section 3.2.1 but is already mentioned in Section 2.4.

17. Provide a citation for the data described in Section 2.4.
18. Italicize *Supplementary Table S1* in Section 2.3.3 for consistency, and arrange the supplementary materials in the order in which they appear in the main text.
19. Clarify what is meant by *Thai mechanistic groups*.
20. Provide an example of a unique study ID for a physical package in Section 2.5.
21. Include a brief description of Section 2.6 before proceeding to its subsections.
22. The citations associated with "n = total number of data points ..." should appear before the equation.
23. The descriptions of the Supplementary Tables do not match those in the main text. For example, Supplementary Table S4 is labeled as *Mapping of Figure Revisions and Justifications*, whereas the main text describes it as containing full cluster properties and statistical distributions.
24. Supplementary Table S4 should be omitted from the main text. Justifications for figure revisions belong in the rebuttal letter, not the manuscript.
25. The formula for θ is not provided.
26. The phrase *"Full DEA results"* is misleading if Table S5 only presents partial results and readers must contact the authors for the complete data.
27. The statement *"This equation has been retained with its mathematical integrity intact..."* is unnecessary and should be removed from the manuscript, as it belongs in the rebuttal letter.
28. Labels such as *n9, n6,* and *N36* in Figure 3 are unnecessary, as the caption already provides sufficient explanation.
29. The arrow labeled *"machine learning"* in the flowchart is unclear. The figure is overly simplified, and the caption does not adequately explain its context.
30. Figure 4B lacks a summary for Cluster 8. Additionally, Figure 4A uses different colors for clusters but does not include a legend identifying them.
31. Several figures contain unnecessary commentary that detracts from readability, reinforcing the impression that the manuscript was revised mainly to address reviewer comments rather than to improve clarity for readers.

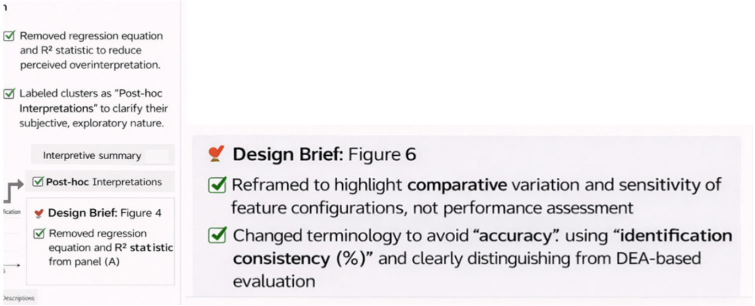

32. Figure 5C contains a typographical error. The number of clusters should simply be listed in the *Cluster* column.

(C) DEA efficiency summary across clusters

**Cluster**	Antibiotic products	DEA efficiency (relative)
Cluster	Strom	0.73
Cluster	Notioxacin	0.96
Cluster	Prepo 400	✓ 1.00
Cluster	Amoxicillin	✓ 1.00
Cluster	Ocpatrir	0.96
Cluster	Ocpatrir	0.96
Cluster	Decisex	✓ 1.00
Cluster	Metrens 400	✓ 1.00
Cluster	Egipa Provolix	0.90

33. Provide introductory descriptions for Sections 3 and 3.2 before their respective subsections.
34. The results in Section 3.2.1 should be summarized in a table.
35. Figure 6A contains five legend entries, whereas Figure 6B shows six plotted lines.
36. Section 4.4 is redundant, as the same information is already presented in Section 2.7.
37. Future directions would be more appropriately placed in the Conclusion (Section 5).
38. *This study proposed and validated a composite informatics framework that integrates image-derived and OCR-based features to evaluate the packaging characteristics of 36 commonly used antibiotic products* **in Thailand**.
39. *I have not seen the* code appendix.
