# Supplementary material for: Image-based identification and DEA-based optimization modeling of antibiotic packaging using unsupervised learning techniques
Source: PLoS One. 2026 Jul 27;21(7):e0354277. doi: 10.1371/journal.pone.0354277 (PMC13405083; doi:10.1371/journal.pone.0354277)
Supplement: Attachment — Submitted filename: Response to Reviewers (Detailed Point-by-Point).pdf [file pone.0354277.s008.pdf]

Response to Reviewers (Detailed)

We thank the reviewers for their careful evaluation and constructive comments. Below we provide a point-by-point response. All page and line numbers refer to the revised tracked manuscript.

Reviewer #1

Comment 1.1: Clarify the study scope and avoid overclaiming clinical deployment.

Response: We agree. The manuscript has been revised to clearly frame the work as an analytical and benchmarking framework rather than a deployable clinical or regulatory decision-support system. This clarification has been added to the Discussion and Conclusion sections.

Reviewer #2

Comment 2.1: Improve reproducibility and transparency of feature definitions.

Response: Addressed. All variables, equations, feature definitions, and analytical steps are now explicitly described in the Methods, with cross-references to Supplementary Tables S1–S8 and the Code Appendix.

Reviewer #4

Comment 31: Figures appear to imply performance benchmarking.

Response: Addressed. All figures (Figures 4–7) have been reframed as exploratory, descriptive, or evaluative. Performance-oriented language has been removed from captions and Results text.

Comment 32: Clarify the balance between figures and tables.

Response: Addressed. Quantitative evidence is now anchored in Table 1 and Supplementary Tables S4–S6, while figures provide structural and interpretive overviews only.

Additional Comment: The DOI in Reference #2 is incorrect.

Response: Thank you for noting this issue. We have verified and corrected the DOI for Reference #2 to the accurate published identifier (https://doi.org/10.1177/2042098614554919). The reference list has been updated accordingly to ensure accuracy and direct accessibility.

All remaining minor comments were addressed through editorial clarification, terminology harmonization, and manuscript hygiene review.
