# Supplementary material for: Image-based identification and DEA-based optimization modeling of antibiotic packaging using unsupervised learning techniques
Source: PLoS One. 2026 Jul 27;21(7):e0354277. doi: 10.1371/journal.pone.0354277 (PMC13405083; doi:10.1371/journal.pone.0354277)
Supplement: Attachment — Submitted filename: PLOS ONE review__.pdf [file pone.0354277.s010.pdf]

The manuscript has improved substantially in terms of writing quality; however, it appears that the revisions were made primarily to satisfy reviewer comments rather than to enhance overall readability for the intended audience. The authors seem to assume a high level of prior familiarity with the topic, which is inconsistent with their claim that the work is novel and represents a first-time application. Several passages read as responses to reviewer critiques and would be more appropriately placed in the rebuttal letter rather than in the main manuscript.

Overall, the study is interesting, and the results offer valuable insights into the future of automated dispensing systems. However, the presentation of the results is often confusing and contains numerous inconsistencies and errors. My specific comments and suggestions are listed below:

1. The DOI in Reference #2 is incorrect. ok
2. Citations in the Introduction should be improved. When making specific claims, the corresponding references should be clearly identified rather than grouped together in a single citation. did not really improve
3. The discussion of DEA in the Introduction remains insufficient. The authors should explain how DEA enables comparative efficiency assessment without assuming predefined functional relationships, and clarify what is meant by such predefined relationships. ok
4. Provide a brief description of the image-based metrics in the Introduction. ok
5. Several terms in Section 2.1 are not defined. ok
6. The term *"steps"* is used in the image, whereas *"stages"* is used in the caption. not ok
7. The term *"mipelee"* in Figure 1 appears to be a typographical error. If not, its meaning should be explained. not ok
8. The flowchart shown below the STEPS in Figure 1 should be explained in the caption. not ok - caption is good but they used A, B, C, D and they are not labeled like that in the figure.
9. Insert a brief description of Section 2.3 before introducing its subsections. ok
10. HSV is not defined when first introduced. not ok
11. In Equation (2), clarify the index over which the summation is taken. ok
12. Figure 2 contains typographical errors: *"incicient," "smooh,"* and *"greeyscale."* not ok. This figure pertains to the methodology rather than the results. The authors should be able to revise this; however, it is unclear why they are unwilling to address the typographical errors.
13. In Figure 2C, clarify what is meant by *"locate"* an algorithm. not ok, A, B, C, D are used in the caption but they are now labeled like that in the figure

14. The captions above the images in Figure 2C are too small to read. Additionally, in the final image, it is unclear which regions correspond to smooth and not smooth areas. The images are overly simplified, and the captions lack sufficient descriptive detail. not ok
15. Provide an explanation of adaptive thresholding in Section 2.3.3. not ok
16. The definition of API appears in Section 3.2.1 but is already mentioned in Section 2.4. ok
17. Provide a citation for the data described in Section 2.4. ok
18. Italicize *Supplementary Table S1* in Section 2.3.3 for consistency, and arrange the supplementary materials in the order in which they appear in the main text. ok
19. Clarify what is meant by *Thai mechanistic groups*. not ok. They did not clarify what are the Thai classifications.
20. Provide an example of a unique study ID for a physical package in Section 2.5. ok
21. Include a brief description of Section 2.6 before proceeding to its subsections. not ok
22. The citations associated with "n = total number of data points ..." should appear before the equation. not ok. they just removed the equations an shorten the discussion.
23. The descriptions of the Supplementary Tables do not match those in the main text. For example, Supplementary Table S4 is labeled as *Mapping of Figure Revisions and Justifications*, whereas the main text describes it as containing full cluster properties and statistical distributions. n/a
24. Supplementary Table S4 should be omitted from the main text. Justifications for figure revisions belong in the rebuttal letter, not the manuscript. n/a
25. The formula for θ is not provided. not ok. Again, they avoided the explanation by removing the formulas.
26. The phrase *"Full DEA results"* is misleading if Table S5 only presents partial results and readers must contact the authors for the complete data. n/a
27. The statement *"This equation has been retained with its mathematical integrity intact..."* is unnecessary and should be removed from the manuscript, as it belongs in the rebuttal letter. ok
28. Labels such as *n9, n6,* and *N36* in Figure 3 are unnecessary, as the caption already provides sufficient explanation. not ok
29. The arrow labeled *"machine learning"* in the flowchart is unclear. The figure is overly simplified, and the caption does not adequately explain its context. not ok

30. Figure 4B lacks a summary for Cluster 8. Additionally, Figure 4A uses different colors for clusters but does not include a legend identifying them. not ok
31. Several figures contain unnecessary commentary that detracts from readability, reinforcing the impression that the manuscript was revised mainly to address reviewer comments rather than to improve clarity for readers. not ok

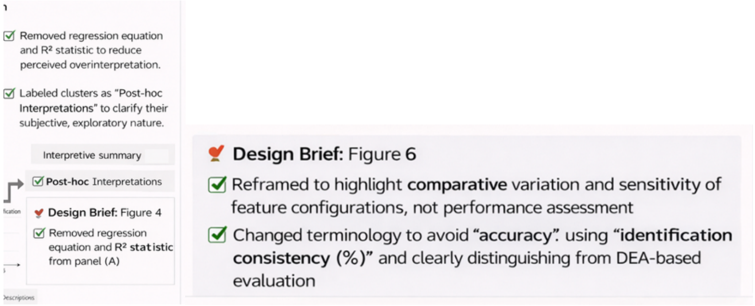

32. Figure 5C contains a typographical error. The number of clusters should simply be listed in the *Cluster* column. not ok. The authors should really revise this because the important part of the Cluster column is the Cluster **number (1, 2, …, 9)**

(C) DEA efficiency summary across clusters

Cluster	Antibiotic products	DEA efficiency (relative)
Cluster	Strom	0.73
Cluster	Notioxacin	0.96
Cluster	Prepo 400	✓ 1.00
Cluster	Amoxicillin	✓ 1.00
Cluster	Ocpatrir	0.96
Cluster	Ocpatrir	0.96
Cluster	Decisex	✓ 1.00
Cluster	Metrens 400	✓ 1.00
Cluster	Egipa Provolix	0.90

33. Provide introductory descriptions for Sections 3 and 3.2 before their respective subsections. not ok
34. The results in Section 3.2.1 should be summarized in a table. not ok

35. Figure 6A contains five legend entries, whereas Figure 6B shows six plotted lines. not ok
36. Section 4.4 is redundant, as the same information is already presented in Section 2.7. not ok
37. Future directions would be more appropriately placed in the Conclusion (Section 5). not ok
38. *This study proposed and validated a composite informatics framework that integrates image-derived and OCR-based features to evaluate the packaging characteristics of 36 commonly used antibiotic products* **in Thailand**.
39. *I have not seen the* code appendix.
