# Supplementary material for: Image-based identification and DEA-based optimization modeling of antibiotic packaging using unsupervised learning techniques
Source: PLoS One. 2026 Jul 27;21(7):e0354277. doi: 10.1371/journal.pone.0354277 (PMC13405083; doi:10.1371/journal.pone.0354277)
Supplement: Attachment — Submitted filename: Response to Reviewers (PLOS ONE)_2026.pdf [file pone.0354277.s011.pdf]

Response to Reviewers (PLOS ONE)

Manuscript Title: Image-Based Identification and DEA-Based Optimization Modeling of Antibiotic Packaging Using Unsupervised Learning Techniques

General Response

We sincerely thank the reviewers and editor for their insightful and constructive comments. We have revised the manuscript strictly in accordance with the reviewers' requests. All modifications were limited to clarification, alignment, and correction of inconsistencies. No new data, analyses, or interpretations were introduced beyond the original scope.

To facilitate review, we provide a point-by-point response with explicit mapping to (i) changes made and (ii) their exact locations in the revised manuscript.

Reviewer 1 (Detailed Point-by-Point Response)

Below we provide a complete response to all 39 reviewer comments, with explicit mapping to manuscript revisions.

Comment 1: DOI in Reference #2 is incorrect

Response: Corrected.
Location: Reference #2

Comment 2: Citations in the Introduction should be improved

Response: Citations were refined to align more closely with specific claims. Where appropriate, grouped citations were retained but clarified through improved sentence structure.

Location: Introduction *(Paragraphs 1–3)*

Comment 3: Insufficient DEA explanation in Introduction

Response: Added clarification explaining that DEA enables comparative efficiency assessment without assuming predefined functional relationships.

Location: Introduction *(DEA paragraph)*

Comment 4: Provide description of image-based metrics

Response: Brief description *added for entropy and PAR*.

Location: Introduction

Comment 5: Undefined terms in Section 2.1

Response: All terms have been defined.

Location: Section 2.1

Comment 6: “steps” vs “stages” inconsistency

Response: Terminology standardized.

Location: Figure 1 and caption

Comment 7: Typo “mipelee” in Figure 1

Response: Corrected.

Location: Figure 1

Comment 8: Flowchart labeling inconsistency

Response: Figure labels aligned with caption (A–D consistency fixed).

Location: Figure 1

Comment 9: Missing Section 2.3 introduction

Response: Introductory paragraph added.

Location: Section 2.3

Comment 10: HSV not defined

Response: Definition added at first occurrence.

Location: Section 2.3

Comment 11: Clarify Equation (2) index

Response: Clarified summation index.

Location: Methods (Equation 2)

Comment 12: Typographical errors in Figure 2

Response: All typographical errors corrected.

Location: Figure 2

Comment 13: Clarify “locate” algorithm in Figure 2C

Response: Caption revised to clarify meaning.

Location: Figure 2C

Comment 14: Figure 2 readability issues

Response: Font sizes increased and descriptions expanded.

Location: Figure 2

Comment 15: Explain adaptive thresholding

Response: Explanation added.

Location: Section 2.3.3

Comment 16: API definition duplication

Response: Removed redundancy.

Location: Section 3.2.1

Comment 17: Missing citation for data

Response: Citation added.

Location: Section 2.4

Comment 18: Formatting of Supplementary Table S1

Response: Standardized formatting and ordering.

Location: Section 2.3.3

Comment 19: Clarify Thai mechanistic groups

Response: Clarified terminology.

Location: Section 2.4

Comment 20: Example of study ID

Response: Example added.

Location: Section 2.5

Comment 21: Missing Section 2.6 introduction

Response: Introductory sentence added.

Location: Section 2.6

Comment 22: Citation placement before equation

Response: Citation placement corrected.

Location: Methods

Comment 23–24: Supplementary Table S4 inconsistency

Response: Removed inappropriate content from main manuscript.

Location: Supplementary Materials

Comment 25: Missing formula for θ

Response: Formula restored and clarified.

Location: Methods

Comment 26: Misleading “Full DEA results”

Response: Revised wording for accuracy.

Location: Supplementary Table S5

Comment 27: Remove rebuttal-like statement

Response: Removed.

Location: Methods

Comment 28: Remove unnecessary labels in Figure 3

Response: Labels simplified.

Location: Figure 3

Comment 29: Unclear “machine learning” arrow

Response: Clarified figure and caption.

Location: Figure 3

Comment 30: Figure 4 issues (Cluster 8 + legend)

Response: Added Cluster 8 summary and legend.

Location: Figure 4

Comment 31: Figures contain unnecessary commentary

Response: Removed redundant annotations and simplified figures.

Location: Figures 3–5

Comment 32: Figure 5C typographical issue

Response: Corrected cluster labeling.

Location: Figure 5

Comment 33: Missing introductions for Sections 3 and 3.2

Response: Introductory sentences added.

Location: Results Section

Comment 34: Table needed for Section 3.2.1

Response: Table added summarizing OCR metrics.

Location: Results (Table 2)

Comment 35: Figure 6 inconsistency (5 vs 6 lines)

Response: Corrected to display six distinct model curves.

Location: Figure 6

Comment 36: Redundancy between Sections 4.4 and 2.7

Response: Redundancy reduced while preserving original structure.

Location: Discussion Section

Comment 37: Future directions placement

Response: Maintained structure with minimal adjustment to avoid redundancy.

Location: Discussion and Conclusion

Comment 38: Statement clarity

Response: Retained as descriptive summary aligned with study scope.

Comment 39: Missing code appendix

Response: Supplementary materials reviewed and clarified; no additional code appendix required for reproducibility as methodological detail is fully provided.

Summary

All 39 reviewer comments have been addressed through targeted revisions. Changes were strictly limited to clarification, correction, and alignment, without introducing new analyses or interpretations.
