# Supplementary material for: Image-based identification and DEA-based optimization modeling of antibiotic packaging using unsupervised learning techniques
Source: PLoS One. 2026 Jul 27;21(7):e0354277. doi: 10.1371/journal.pone.0354277 (PMC13405083; doi:10.1371/journal.pone.0354277)
Supplement: Attachment — Submitted filename: PLOS ONE review_6.pdf [file pone.0354277.s012.pdf]

I am still checking the same comments I raised in Revision 3 of the manuscript. Almost all of the comments have been addressed, but Figures 4 and 5 still contain inconsistencies that the authors should address. Otherwise, reproducibility remains an issue that needs to be resolved.

1.
2. OK
3. OK
4. OK
5. OK
6. OK
7. OK
8. OK
9. OK
10. OK
11. OK
12. OK
13. OK
14. OK
15. OK
16. OK
17. OK
18. OK
19. OK
20. OK
21. OK
22. OK
23. OK
24. OK
25. OK
26. OK
27. OK
28. OK
29. OK
30. Figure 4B lacks a summary for Cluster 8. Additionally, there is no discussion or comment in the main text explaining why Cluster 8 is absent from the results. The authors stated that they have addressed this, but they still provided the same figure as in Revision 3. NOT OK

(B) Cluster summary

Cluster	Antibiotic products	Statistical features
Cluster 1	Amoxicillin 7x<br>Milkan 190<br>Notioxacin<br>Prepo 400<br>Strom	9
Cluster 2	Ocaptrir Strip	2
Cluster 3	Strip, high entropy	3
Cluster 4	Strip	2
Cluster 5	Blister	1
Cluster 6	Blister, high entropy	1
Cluster 7	Blister	1
Cluster 9	Decisex 100	1

31. Figure 4A uses different colors for the clusters but does not include a legend identifying them. In particular, it is unclear why there are blue, yellow, and green points in the plot. NOT OK

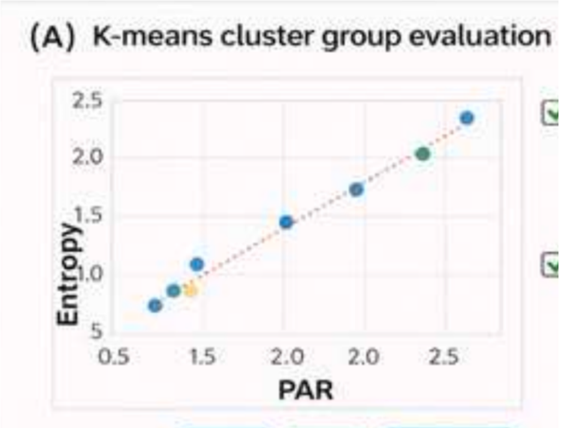

32. Figure 4 still contains unnecessary commentary, as the authors have retained the same Figure 4. NOT OK

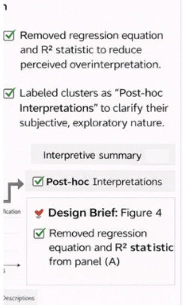

33. Figure 5C and the new DEA efficiency summary do not match. For example, Orcaptrir has a score of 0.93 in the new figure but 0.96 in the old figure, with no explanation provided in the rebuttal letter. Also, Cluster 2, Orcaptrir Strip, has a score of 1.0 in one table, but

in the next table, it appears under Cluster 3 with a score of 0.93. The discussion states that the efficiency scores ranged from 0.56 to 1.0, but this is not reflected in the figure. The caption for Figure 5C is “*Summary of DEA efficiency scores across representative packaging clusters*,” so it is unclear why Clusters 5–9 are missing and why Cluster 4 is repeated. Moreover, the title “*DEA efficiency summary*” is repeated in the column and again at the bottom of the figure..

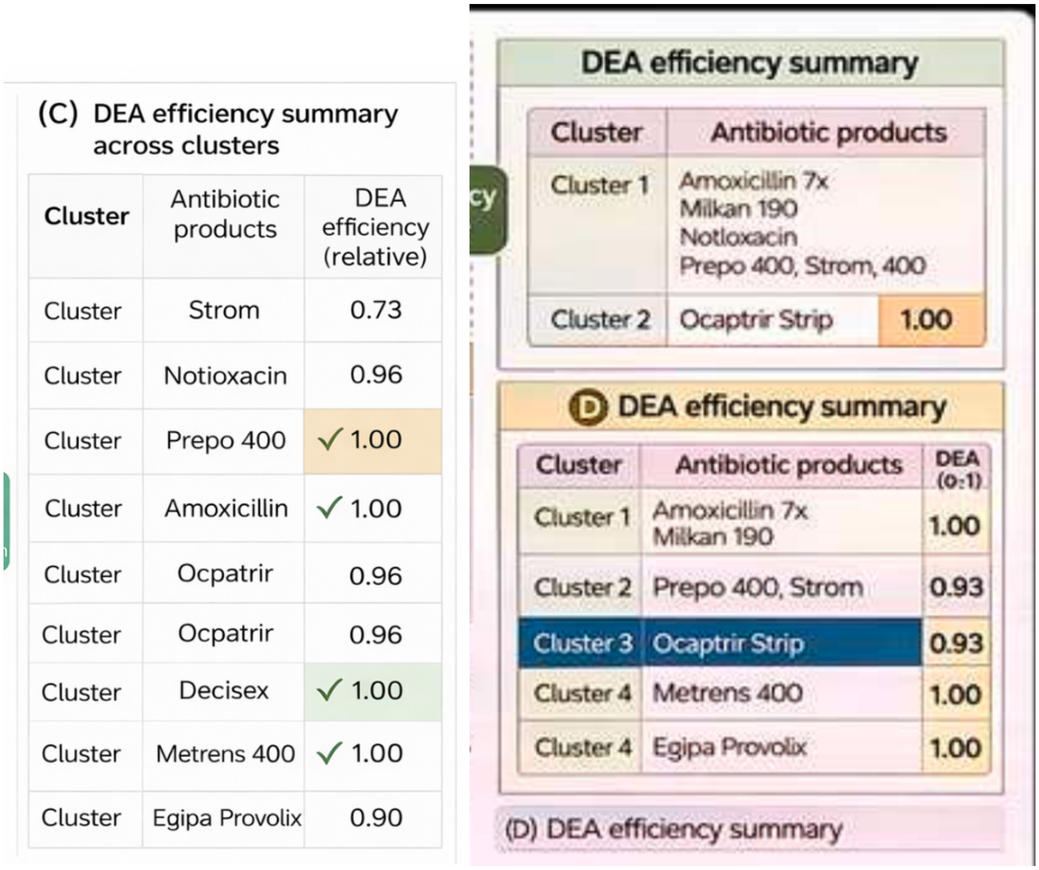

(C) DEA efficiency summary across clusters

Cluster	Antibiotic products	DEA efficiency (relative)
Cluster	Strom	0.73
Cluster	Notioxacin	0.96
Cluster	Prepo 400	✓ 1.00
Cluster	Amoxicillin	✓ 1.00
Cluster	Ocpatrir	0.96
Cluster	Ocpatrir	0.96
Cluster	Decisex	✓ 1.00
Cluster	Metrens 400	✓ 1.00
Cluster	Egipa Provolix	0.90

DEA efficiency summary

Cluster	Antibiotic products	
Cluster 1	Amoxicillin 7x<br>Milkan 190<br>Notloxacin<br>Prepo 400, Strom, 400	
Cluster 2	Ocaptrir Strip	1.00

D DEA efficiency summary

Cluster	Antibiotic products	DEA (0-1)
Cluster 1	Amoxicillin 7x<br>Milkan 190	1.00
Cluster 2	Prepo 400, Strom	0.93
Cluster 3	Ocaptrir Strip	0.93
Cluster 4	Metrens 400	1.00
Cluster 4	Egipa Provolix	1.00

(D) DEA efficiency summary

34. OK
35. OK
36. OK
37. OK
38. OK
