# Supplementary material for: Image-based identification and DEA-based optimization modeling of antibiotic packaging using unsupervised learning techniques
Source: PLoS One. 2026 Jul 27;21(7):e0354277. doi: 10.1371/journal.pone.0354277 (PMC13405083; doi:10.1371/journal.pone.0354277)
Supplement: Attachment — Submitted filename: Response to Reviewers R2.docx [file pone.0354277.s006.docx]

# **Response to Reviewers (Hybrid Format)**

This document presents a **hybrid response format** combining a concise narrative response with a structured Reviewer’s Comment-to-Revision Mapping Table. This approach was adopted to clearly demonstrate both conceptual engagement with reviewer critiques and precise implementation of requested revisions, in line with PLOS ONE editorial expectations.

## **Part I. Narrative Response to Reviewers**

We sincerely thank the **Academic Editor and Reviewer #4** for their detailed and constructive feedback. In response, we have undertaken a **substantial revision** of the manuscript that goes beyond technical clarification. The revision focused on (i) reframing the study motivation and novelty, (ii) improving methodological coherence and interpretability, and (iii) aligning section placement with reviewer instructions.

**Major Revision Highlights**

- The **Introduction was fully rewritten** using a problem-driven framing that explicitly motivates the need for packaging-level antibiotic identification within automated pharmacy workflows.
- The **Methods section was re-ordered and reframed** to clarify the purpose of each analytical component (image features, clustering, and DEA) before technical implementation.
- **Packaging Area Ratio (PAR)** was repositioned and expanded as a normalization and design-relevant metric within Methods.
- **Data Envelopment Analysis (DEA)** was explicitly reframed as a decision-oriented evaluation layer rather than a classifier, clarifying methodological novelty.
- **Study limitations were relocated** to appear before the Results section, in direct accordance with reviewer instructions.
- **Figure 5 was revised** to improve interpretability and to explicitly communicate the role of DEA as an **evaluation layer rather than a predictive model**. Panel labeling, typographical elements, and captions were refined in direct response to Reviewer #4.
- **Figure 4 was refined** to clarify the **exploratory role of K-means clustering**, with cluster descriptions explicitly presented as **post-hoc interpretations** rather than supervised labels.
- **Figure 6 was revised** to clarify its role as a **comparative sensitivity and variation analysis**, not a performance evaluation. Terminology, panel titles, and captions were refined to avoid accuracy-based interpretation and to ensure conceptual distinction from DEA benchmarking results (Figure 5).
- **Figure 7 was refined** to clarify that it provides a **descriptive, conceptual summary of relative feature emphasis** in API identification. The figure title and caption were revised to explicitly state that the reported percentages represent normalized, illustrative summaries rather than statistically learned feature importance or performance-based weighting.

All revisions are reflected in the tracked-changes manuscript and summarized in the mapping table below.

## **Part II. Reviewer’s Comment-to-Revision Mapping Table**

| Reviewer Comment / Concern | Action Taken | Location in Revised Manuscript |
| --- | --- | --- |
| Introduction needs to be rewritten to clearly motivate the study | Introduction fully rewritten with problem-driven framing, literature positioning, and explicit novelty | Section 1 (Introduction) |
| Use of terms such as “urgent” or “critical” insufficient to justify study | Added system-level rationale linking automation and dispensing workflow risks | Section 1 (Introduction) |
| Insufficient discussion of related literature | Prior pill-level and OCR-based studies contextualized with explicit limitations | Section 1 (Introduction) |
| Novelty not clearly stated in the Introduction | Three explicit novelty points articulated within Introduction | Section 1 (Introduction) |
| Lack of coherent flow between manuscript sections | Added Methods Overview and re-ordered methodological narrative (why → how) | Section 2 (Methods Overview) |
| PAR discussion misplaced in Results | PAR reframed and relocated as normalization metric | Section 2.3.1 |
| K-means clustering mentioned without explanation | Added conceptual justification for unsupervised clustering choice | Section 2.6.1 |
| DEA novelty unclear and underdeveloped | DEA reframed as evaluation and optimization layer | Section 2.6.2 |
| Study limitations placed incorrectly | Limitations relocated before Results section | Section 2.7 |
| Figures and captions require improved clarity | Figures retained and captions refined for interpretability; Figure 5 revised to emphasize DEA as an evaluation layer (not a classifier), Figure 4 refined to clarify the exploratory nature of K-means clustering with post-hoc interpretation of clusters, Figure 6 reframed to present comparative variation and sensitivity of feature–output relationships rather than performance evaluation, and Figure 7 clarified as a descriptive, conceptual summary of relative feature emphasis rather than learned importance | Figures 4–7 |
| Typographical errors in Figure 5 | Errors corrected; panel labeling and visual hierarchy refined to improve interpretability | Figure 5 |

## **Closing Statement**

We believe that this hybrid response format demonstrates both thorough engagement with reviewer feedback and transparent implementation of requested revisions. We respectfully submit the revised manuscript for reconsideration and thank the Editor and Reviewers for the opportunity to improve our work.

Sincerely,

Phakdee Sukpornsawan (First author),

**Suthabordee Muongmee (Corresponding author)**
