# Supplementary material for: Image-based identification and DEA-based optimization modeling of antibiotic packaging using unsupervised learning techniques
Source: PLoS One. 2026 Jul 27;21(7):e0354277. doi: 10.1371/journal.pone.0354277 (PMC13405083; doi:10.1371/journal.pone.0354277)
Supplement: Attachment — Submitted filename: Response_R4_map to_Track_Changes.docx [file pone.0354277.s009.docx]

# **Response to Reviewers (R4)**

Structured by Manuscript Sections (Tabular Version)

**Manuscript ID:** PONE-D-25-36293R4

**Title:** Image-Based Identification and DEA-Based Optimization Modeling of Antibiotic Packaging Using Unsupervised Learning Techniques

## **Purpose of this Version**

This table-form response summarizes the revisions performed in Revision 4 (R4), organized according to the manuscript structure. It is intended to provide the Academic Editor with a clear, section-by-section overview of how reviewer concerns were addressed, without introducing new claims or analyses.

### **Section-by-Section Response Matrix**

| Manuscript Section | Reviewer / Editor Concern | Revision Implemented (R4) | Evidence in Revised Manuscript |
| --- | --- | --- | --- |
| Overall Manuscript | Manuscript reads as rebuttal-driven and lacks cohesion | Rewritten with a reader-first narrative; rebuttal-style phrasing removed; clear separation of descriptive and interpretive sections | Introduction–Conclusion rewritten; tone neutralized throughout |
| Introduction | Unclear framing and reader expectations | Reframed to emphasize packaging-level identification and descriptive analytical scope; over-claiming removed | Introduction, final paragraphs |
| Methods (General) | Blurred methodological roles and missing definitions | Explicit role separation for all analytical components; standardized terminology | Methods Sections 2.2–2.6 |
| Methods – Feature Extraction | Insufficient clarity on image-derived metrics | Detailed descriptions retained in Methods; extended procedural details moved to Supplementary Methods | Methods 2.3; Supplementary Methods S1–S2 |
| Methods – Clustering | Ambiguity in clustering validation | Exploratory role clarified; internal validity metric documented without influencing results | Methods 2.5; Supplementary Methods S3 |
| Methods – DEA | DEA potentially interpreted as predictive or optimization model | DEA reframed explicitly as a comparative evaluation layer; no optimality or prediction claims | Methods 2.6; Discussion 4.3 |
| Results (General) | Results intermixed with interpretation | Results rewritten to report observed patterns and descriptive statistics only | Results Sections 3.1–3.5 |
| Results – Tables/Figures | Tables and figures unclear or misplaced | All figures assigned single narrative roles; captions rewritten; placement corrected | Figures 1–7; Table 1 |
| Discussion | Defensive tone and scope overlap | Discussion rewritten as interpretation-only; limitations stated neutrally | Discussion Sections 4.1–4.4 |
| Conclusion | Overlap with Results and Discussion | Conclusion condensed to high-level synthesis without repetition or new claims | Conclusion section |
| Abstract | Potential misalignment with revised manuscript | Abstract reviewed and retained without substantive modification, as it accurately reflected the revised content | Abstract |
| Supplementary Materials | Reproducibility and methodological transparency | Supplementary Methods and Tables provided to document procedures, variables, DEA configuration, and dataset composition | Supplementary Methods S1–S5; Tables S1–S3 |
| References / Citations | Risk of inconsistency after revision | Full reference-to-manuscript mapping performed; numeric citation baseline locked | All sections; References |

### **Closing Statement**

All revisions in R4 were limited to restructuring, clarification, and documentation. No new data, experiments, or analytical outcomes were introduced. This structured response is fully consistent with the revised manuscript and supplementary materials.
