# Supplementary material for: Image-based identification and DEA-based optimization modeling of antibiotic packaging using unsupervised learning techniques
Source: PLoS One. 2026 Jul 27;21(7):e0354277. doi: 10.1371/journal.pone.0354277 (PMC13405083; doi:10.1371/journal.pone.0354277)
Supplement: Attachment — Submitted filename: Response to Reviewer_R6.docx [file pone.0354277.s013.docx]

# **Response to Reviewer (Final Version)**

Reviewer Comment 30: Figure 4B lacks a summary for Cluster 8. Additionally, there is no discussion/comments in the main text that talks about why there is NO Cluster 8 in their results.

**Response:** We thank the reviewer for identifying this issue. Figure 4 has been revised to explicitly include Cluster 8 within the cluster summary panels. The revised figure now presents all identified clusters consistently, and the corresponding Results section has been checked for consistency with the figure.

Reviewer Comment 31: Figure 4A uses different colors for clusters but does not include a legend.

**Response:** A cluster legend has been retained and verified in Figure 4A to ensure that all cluster colors are clearly identified and consistently represented throughout the figure.

Reviewer Comment 32: Figure 4 still contains unnecessary commentary.

**Response:** Interpretive descriptors previously included in Figure 4 (e.g., entropy-based qualitative labels) have been removed. The revised figure now presents descriptive clustering results only, without additional interpretive commentary.

Reviewer Comment 33: Figure 5C and the DEA efficiency summary do not match.

**Response:** Figure 5 has been revised and cross-checked against the DEA source data. Duplicate DEA summary panels were removed, cluster assignments were verified, and DEA efficiency values were synchronized across the figure, manuscript text, and supplementary materials to ensure consistency.
