# Supplementary material for: Image-based identification and DEA-based optimization modeling of antibiotic packaging using unsupervised learning techniques
Source: PLoS One. 2026 Jul 27;21(7):e0354277. doi: 10.1371/journal.pone.0354277 (PMC13405083; doi:10.1371/journal.pone.0354277)
Supplement: Peer review history

# Peer review: Decision Letter 0

**DOI:** 10.1371/journal.pone.0354277.r001

**Authors:** Renier Mendoza

17 Sep 2025

PONE-D-25-36293

Image-Based Identification and Optimization Modeling of Antibiotic Packaging Using Unsupervised Learning Techniques

PLOS ONE

Dear Dr. Muongmee,

Thank you for submitting your manuscript to PLOS ONE. After careful consideration, we feel that it has merit but does not fully meet PLOS ONE’s publication criteria as it currently stands. Therefore, we invite you to submit a revised version of the manuscript that addresses the points raised during the review process.

The manuscript presents a relevant study on applying image processing, OCR, and machine learning to analyze antibiotic packaging in Thailand, with potential contributions to medication safety. However, several areas need further development. The methodology requires clearer descriptions of algorithms, models, and equations, along with appropriate validation to support the reported results. Improvements in writing, formatting, figures, and citations would also strengthen readability. Providing access to data or code, comparing results more critically with related studies, and outlining future directions would enhance the paper’s clarity, rigor, and overall impact. Please refer to the reviewers' comments below for the complete recommendations.

Please submit your revised manuscript by Nov 01 2025 11:59PM. If you will need more time than this to complete your revisions, please reply to this message or contact the journal office at plosone@plos.org. When you're ready to submit your revision, log on to https://www.editorialmanager.com/pone/ and select the 'Submissions Needing Revision' folder to locate your manuscript file.

Please include the following items when submitting your revised manuscript:

- A rebuttal letter that responds to each point raised by the academic editor and reviewer(s). You should upload this letter as a separate file labeled 'Response to Reviewers'.
- A marked-up copy of your manuscript that highlights changes made to the original version. You should upload this as a separate file labeled 'Revised Manuscript with Track Changes'.
- An unmarked version of your revised paper without tracked changes. You should upload this as a separate file labeled 'Manuscript'.

If you would like to make changes to your financial disclosure, please include your updated statement in your cover letter. Guidelines for resubmitting your figure files are available below the reviewer comments at the end of this letter.

If applicable, we recommend that you deposit your laboratory protocols in protocols.io to enhance the reproducibility of your results. Protocols.io assigns your protocol its own identifier (DOI) so that it can be cited independently in the future. For instructions see: https://journals.plos.org/plosone/s/submission-guidelines#loc-laboratory-protocols. Additionally, PLOS ONE offers an option for publishing peer-reviewed Lab Protocol articles, which describe protocols hosted on protocols.io. Read more information on sharing protocols at https://plos.org/protocols?utm_medium=editorial-email&utm_source=authorletters&utm_campaign=protocols.

We look forward to receiving your revised manuscript.

Kind regards,

Renier Mendoza

Academic Editor

PLOS ONE

Journal Requirements:

When submitting your revision, we need you to address these additional requirements.

1. Please ensure that your manuscript meets PLOS ONE's style requirements, including those for file naming. The PLOS ONE style templates can be found at

https://journals.plos.org/plosone/s/file?id=wjVg/PLOSOne_formatting_sample_main_body.pdf and https://journals.plos.org/plosone/s/file?id=ba62/PLOSOne_formatting_sample_title_authors_affiliations.pdf

2. Please provide additional details regarding participant consent. In the ethics statement in the Methods and online submission information, please ensure that you have specified (1) whether consent was informed and (2) what type you obtained (for instance, written or verbal, and if verbal, how it was documented and witnessed). If your study included minors, state whether you obtained consent from parents or guardians. If the need for consent was waived by the ethics committee, please include this information.

If you are reporting a retrospective study of medical records or archived samples, please ensure that you have discussed whether all data were fully anonymized before you accessed them and/or whether the IRB or ethics committee waived the requirement for informed consent. If patients provided informed written consent to have data from their medical records used in research, please include this information.

3. Please note that PLOS One has specific guidelines on code sharing for submissions in which author-generated code underpins the findings in the manuscript. In these cases, we expect all author-generated code to be made available without restrictions upon publication of the work. Please review our guidelines at https://journals.plos.org/plosone/s/materials-and-software-sharing#loc-sharing-code and ensure that your code is shared in a way that follows best practice and facilitates reproducibility and reuse.

4. We note that your Data Availability Statement is currently as follows: [All relevant data are within the manuscript and its Supporting Information files.]

Please confirm at this time whether or not your submission contains all raw data required to replicate the results of your study. Authors must share the “minimal data set” for their submission. PLOS defines the minimal data set to consist of the data required to replicate all study findings reported in the article, as well as related metadata and methods (https://journals.plos.org/plosone/s/data-availability#loc-minimal-data-set-definition).

For example, authors should submit the following data:

- The values behind the means, standard deviations and other measures reported;

- The values used to build graphs;

- The points extracted from images for analysis.

Authors do not need to submit their entire data set if only a portion of the data was used in the reported study.

If your submission does not contain these data, please either upload them as Supporting Information files or deposit them to a stable, public repository and provide us with the relevant URLs, DOIs, or accession numbers. For a list of recommended repositories, please see https://journals.plos.org/plosone/s/recommended-repositories.

If there are ethical or legal restrictions on sharing a de-identified data set, please explain them in detail (e.g., data contain potentially sensitive information, data are owned by a third-party organization, etc.) and who has imposed them (e.g., an ethics committee). Please also provide contact information for a data access committee, ethics committee, or other institutional body to which data requests may be sent. If data are owned by a third party, please indicate how others may request data access.

5. When completing the data availability statement of the submission form, you indicated that you will make your data available on acceptance. We strongly recommend all authors decide on a data sharing plan before acceptance, as the process can be lengthy and hold up publication timelines. Please note that, though access restrictions are acceptable now, your entire data will need to be made freely accessible if your manuscript is accepted for publication. This policy applies to all data except where public deposition would breach compliance with the protocol approved by your research ethics board. If you are unable to adhere to our open data policy, please kindly revise your statement to explain your reasoning and we will seek the editor's input on an exemption. Please be assured that, once you have provided your new statement, the assessment of your exemption will not hold up the peer review process.

6. Please upload a new copy of Figures, 1, 2, 4, 5, 6, 7, 8, and 9 as the details are not clear. Please follow the link for more information: https://blogs.plos.org/plos/2019/06/looking-good-tips-for-creating-your-plos-figures-graphics/

If the reviewer comments include a recommendation to cite specific previously published works, please review and evaluate these publications to determine whether they are relevant and should be cited. There is no requirement to cite these works unless the editor has indicated otherwise.

[Note: HTML markup is below. Please do not edit.]

Reviewers' comments:

Reviewer's Responses to Questions

**Comments to the Author**

1. Is the manuscript technically sound, and do the data support the conclusions?

The manuscript must describe a technically sound piece of scientific research with data that supports the conclusions. Experiments must have been conducted rigorously, with appropriate controls, replication, and sample sizes. The conclusions must be drawn appropriately based on the data presented.

Reviewer #1: No

Reviewer #2: Yes

Reviewer #3: Partly

**********

2. Has the statistical analysis been performed appropriately and rigorously?

Reviewer #1: No

Reviewer #2: Yes

Reviewer #3: N/A

**********

3. Have the authors made all data underlying the findings in their manuscript fully available?

The PLOS Data policy requires authors to make all data underlying the findings described in their manuscript fully available without restriction, with rare exception (please refer to the Data Availability Statement in the manuscript PDF file). The data should be provided as part of the manuscript or its supporting information, or deposited to a public repository. For example, in addition to summary statistics, the data points behind means, medians and variance measures should be available. If there are restrictions on publicly sharing data—e.g. participant privacy or use of data from a third party—those must be specified.

Reviewer #1: No

Reviewer #2: No

Reviewer #3: Yes

**********

4. Is the manuscript presented in an intelligible fashion and written in standard English?

PLOS ONE does not copyedit accepted manuscripts, so the language in submitted articles must be clear, correct, and unambiguous. Any typographical or grammatical errors should be corrected at revision, so please note any specific errors here.

Reviewer #1: Yes

Reviewer #2: Yes

Reviewer #3: Yes

**********

5. Review Comments to the Author

Please use the space provided to explain your answers to the questions above. You may also include additional comments for the author, including concerns about dual publication, research ethics, or publication ethics. (Please upload your review as an attachment if it exceeds 20,000 characters)

Reviewer #1: The manuscript explores the application of image processing, optical character recognition (OCR), and unsupervised learning techniques to analyze the packaging of antibiotic products distributed in Thailand. The study’s stated goal is to develop a method for improving medication safety through automated analysis of packaging characteristics using clustering (via K-means) and efficiency assessment (via Data Envelopment Analysis, DEA).

While the topic is relevant and potentially impactful in pharmacy automation and drug safety, the manuscript suffers from several critical shortcomings in methodology, clarity of writing, statistical rigor, and presentation. At its current stage, the paper does not meet the standards for publication in PLOS ONE.

1. Writing Quality and Presentation

The manuscript requires substantial improvements in writing and formatting:

There are multiple typographical errors, such as:

"traceabilit" instead of "traceability" (page 1)

"recognizability" instead of "recognizability" (page 5)

"k-mean" instead of "k-means" in graph labels

There are numerous formatting inconsistencies, including sudden changes in font style and size, making the manuscript visually disjointed and difficult to follow.

The writing is often verbose and repetitive, particularly in the introduction and methods sections. Key methodological steps are not always clearly delineated or logically presented.

Terminology such as "identification" is overused and incorrectly applied to unsupervised methods. The paper should clearly distinguish between clustering, classification, and detection.

2. Reproducibility and Transparency

There is no publicly accessible code, workflow diagram, or supplementary material that would enable replication of the methods.

The authors state that the data are available, but there are no links or repositories provided.

3. Scientific Soundness and Methodological Issues

The methodology section is underdeveloped and lacks sufficient clarity. Many technical terms are introduced without adequate explanation or justification (e.g., entropy smoothing, histogram stretching, feature vector composition).

The paper heavily relies on unsupervised clustering (K-means) but makes repeated claims about classification accuracy, which is misleading. Clustering is not equivalent to classification, and the manuscript seemingly combines these concepts. This terminological confusion undermines the validity of reported performance claims (e.g., "accuracy > 95%" from unsupervised models without labeled data or classification evaluation metrics).

There is no use of supervised machine learning models (e.g., Random Forests, SVM, CNN) that would be standard for a classification task involving labeled packaging data. This weakens the empirical support for the claimed performance.

The DEA model is described mathematically, but it lacks interpretability and practical context. Its integration into the analysis is unclear.

4. Statistical and Validation Weaknesses

There is no cross-validation or external validation, which raises serious concerns about overfitting and generalizability, especially considering the small dataset of 36 antibiotic products.

Regression models with very low explanatory power (e.g., R² = 0.12) are still interpreted for insight, which is statistically inappropriate.

No confusion matrices, F1 scores, precision/recall, or other standard classification metrics are presented.

Overall Assessment

Despite the relevance of the topic and the potential utility of integrating image processing and OCR in pharmacy systems, this manuscript does not meet the technical and editorial standards required for publication. Major revisions would be needed to:

Clearly distinguish between clustering and classification,

Strengthen the methodology with supervised models and validation,

Improve statistical rigor,

Correct typographical and formatting issues,

Sharpen the clarity and coherence of the writing.

At this time, however, the manuscript may require more substantial revision than can typically be accommodated through peer review. For this reason, I recommend that the authors consider revising the manuscript thoroughly before resubmitting. I hope this feedback will be helpful in strengthening the paper.

Reviewer #2: - Best efforts were made in data acquisition.

- In Section 2.1 Step 3, specify the machine learning algorithms used.

- In Section 2.2 item #1, what was the pre-trained object detection model used? Please elaborate more on this.

- Please give more details/description about the Canny edge filter and Global Otsu thresholding and provide proper citations

- In Section 2.3, specify the distance metrics tested.

- Explain and describe Equation 1 — what is H? p(i)? What is the index of summation and the lower and upper limit of summation?

- In the last paragraph of Section 2.3, why did you choose F-score as your predictive performance? Moreover, is the 11% improvement in F-score from your results, or from reference [28]?

- In Table 1, how much does each feature affect the classification accuracy?

- Give proper citation to Tesseract OCR

- Properly cite Thai FDA registry;

- Properly cite DEA by Charnes et al.

- Fix equation (3) and its constraints

- Maximize E_0 with respect to what?

- The first constraint must be less than or equal to what?

- Second constraint: w_j, v_i >= 0

- Cite your reference for equation 3.

- There is room for improvement for the figures.

- Where can we access the data used in the study?

Minor comments:

1. Correct misspelled words in text and in images.

2. Please use high resolution figures and fix the font size.

3. Some section titles can be improved.

Reviewer #3: The introduction repeats well-known facts about antibiotic misuse without tightly linking them to the image-analysis focus.

International comparisons (e.g., U.S. data) are mentioned but not integrated meaningfully with the Thai dataset.

The methods section is very detailed about imaging conditions (lux, color calibration, equipment), but some parts feel excessive and could overwhelm readers while still leaving gaps (e.g., no explanation of why only 36 antibiotics were selected).

The description of pre-processing and clustering lacks validation: there is no mention of cross-validation, external test sets, or reproducibility checks.

The results are overloaded with regression equations (R² values reported even when correlations are weak, e.g., R² = 0.12) without critical interpretation.

The discussion mostly restates results instead of critically comparing them with related studies.

No future work or roadmap is clearly outlined in the conclusion section.

**********

6. PLOS authors have the option to publish the peer review history of their article (what does this mean?). If published, this will include your full peer review and any attached files.

If you choose “no”, your identity will remain anonymous but your review may still be made public.

**Do you want your identity to be public for this peer review?**  For information about this choice, including consent withdrawal, please see our Privacy Policy.

Reviewer #1: No

Reviewer #2: No

Reviewer #3: No

**********

[NOTE: If reviewer comments were submitted as an attachment file, they will be attached to this email and accessible via the submission site. Please log into your account, locate the manuscript record, and check for the action link "View Attachments". If this link does not appear, there are no attachment files.]

While revising your submission, please upload your figure files to the Preflight Analysis and Conversion Engine (PACE) digital diagnostic tool, https://pacev2.apexcovantage.com/. PACE helps ensure that figures meet PLOS requirements. To use PACE, you must first register as a user. Registration is free. Then, login and navigate to the UPLOAD tab, where you will find detailed instructions on how to use the tool. If you encounter any issues or have any questions when using PACE, please email PLOS at figures@plos.org. Please note that Supporting Information files do not need this step.

# Author comment: Author response to Decision Letter 1

**DOI:** 10.1371/journal.pone.0354277.r002

4 Oct 2025

Response to Reviewers

We would like to sincerely thank the Academic Editor and the Reviewers for their thoughtful and constructive comments. We have carefully revised the manuscript in response to these suggestions.

Below, we provide a point-by-point rebuttal letter. Reviewer comments are presented in italics, followed by our detailed responses and the corresponding locations in the revised manuscript.

For clarity, we also include the Reviewer Comment-to-Revision Mapping Table (Full Coverage) (see attached document), which summarizes all comments, manuscript locations, and revisions implemented.

We hope these revisions adequately address all reviewer concerns. We are deeply grateful for the constructive feedback, which has substantially improved the clarity, rigor, and overall quality of the manuscript.

Attachment Submitted filename: Response to Reviewers.pdf

# Editor report: Decision Letter 1

**DOI:** 10.1371/journal.pone.0354277.r003

**Authors:** Renier Mendoza

7 Oct 2025

PONE-D-25-36293R1

Image-Based Identification and DEA-Based Optimization Modeling of Antibiotic Packaging Using Unsupervised Learning Techniques

PLOS ONE

Dear Dr. Muongmee,

Thank you for submitting your manuscript to PLOS ONE. After careful consideration, we feel that it has merit but does not fully meet PLOS ONE’s publication criteria as it currently stands. Therefore, we invite you to submit a revised version of the manuscript that addresses the points raised during the review process.

Please refer to my comments/suggestions below.

Please submit your revised manuscript by Nov 21 2025 11:59PM. If you will need more time than this to complete your revisions, please reply to this message or contact the journal office at plosone@plos.org. When you're ready to submit your revision, log on to https://www.editorialmanager.com/pone/ and select the 'Submissions Needing Revision' folder to locate your manuscript file.

Please include the following items when submitting your revised manuscript:

- A rebuttal letter that responds to each point raised by the academic editor and reviewer(s). You should upload this letter as a separate file labeled 'Response to Reviewers'.
- A marked-up copy of your manuscript that highlights changes made to the original version. You should upload this as a separate file labeled 'Revised Manuscript with Track Changes'.
- An unmarked version of your revised paper without tracked changes. You should upload this as a separate file labeled 'Manuscript'.

If you would like to make changes to your financial disclosure, please include your updated statement in your cover letter. Guidelines for resubmitting your figure files are available below the reviewer comments at the end of this letter.

If applicable, we recommend that you deposit your laboratory protocols in protocols.io to enhance the reproducibility of your results. Protocols.io assigns your protocol its own identifier (DOI) so that it can be cited independently in the future. For instructions see: https://journals.plos.org/plosone/s/submission-guidelines#loc-laboratory-protocols. Additionally, PLOS ONE offers an option for publishing peer-reviewed Lab Protocol articles, which describe protocols hosted on protocols.io. Read more information on sharing protocols at https://plos.org/protocols?utm_medium=editorial-email&utm_source=authorletters&utm_campaign=protocols.

We look forward to receiving your revised manuscript.

Kind regards,

Renier Mendoza

Academic Editor

PLOS ONE

Journal Requirements:

If the reviewer comments include a recommendation to cite specific previously published works, please review and evaluate these publications to determine whether they are relevant and should be cited. There is no requirement to cite these works unless the editor has indicated otherwise.

Additional Editor Comments:

Please resubmit your manuscript together with a detailed, per-reviewer response addressing each comment and recommendation from the referees. The current Reviewer’s Comment-to-Revision Mapping Table is difficult to follow. You may still include it, but kindly provide a separate file that clearly presents your point-by-point responses organized by reviewer.

[Note: HTML markup is below. Please do not edit.]

[NOTE: If reviewer comments were submitted as an attachment file, they will be attached to this email and accessible via the submission site. Please log into your account, locate the manuscript record, and check for the action link "View Attachments". If this link does not appear, there are no attachment files.]

While revising your submission, please upload your figure files to the Preflight Analysis and Conversion Engine (PACE) digital diagnostic tool, https://pacev2.apexcovantage.com/. PACE helps ensure that figures meet PLOS requirements. To use PACE, you must first register as a user. Registration is free. Then, login and navigate to the UPLOAD tab, where you will find detailed instructions on how to use the tool. If you encounter any issues or have any questions when using PACE, please email PLOS at figures@plos.org. Please note that Supporting Information files do not need this step.

# Author comment: Author response to Decision Letter 2

**DOI:** 10.1371/journal.pone.0354277.r004

21 Oct 2025

Rebuttal Letter to the Academic Editor and Reviewers

Manuscript ID: PONE-D-25-36293R1

Title: Image-Based Identification and DEA-Based Optimization Modeling of Antibiotic Packaging Using Unsupervised Learning Techniques

Cover Note

Dear Dr. Renier Mendoza and Esteemed Reviewers,

We sincerely thank the Academic Editor and the Reviewers for their thoughtful and constructive feedback. We have carefully revised the manuscript to address every comment in detail. The manuscript has been substantially improved in clarity, methodological rigor, and contextual interpretation.

We believe these revisions fully address all reviewer concerns and align the paper with PLOS ONE publication standards.

Thank you for your time and consideration.

Sincerely,

Ms. Suthabordee Muongmee

Corresponding Author

Faculty of Pharmaceutical Science, Burapha University

Email: suthabordee@go.buu.ac.th

Attachment Submitted filename: Rebuttal_Letter_221025.pdf

# Peer review: Decision Letter 2

**DOI:** 10.1371/journal.pone.0354277.r005

**Authors:** Renier Mendoza

19 Dec 2025

PONE-D-25-36293R2

Image-Based Identification and DEA-Based Optimization Modeling of Antibiotic Packaging Using Unsupervised Learning Techniques

PLOS One

Dear Dr. Muongmee,

Thank you for submitting your manuscript to PLOS ONE. After careful consideration, we feel that it has merit but does not fully meet PLOS ONE’s publication criteria as it currently stands. Therefore, we invite you to submit a revised version of the manuscript that addresses the points raised during the review process.

Please submit your revised manuscript by Feb 02 2026 11:59PM. If you will need more time than this to complete your revisions, please reply to this message or contact the journal office at plosone@plos.org. When you're ready to submit your revision, log on to https://www.editorialmanager.com/pone/ and select the 'Submissions Needing Revision' folder to locate your manuscript file.

Please include the following items when submitting your revised manuscript:

- A letter that responds to each point raised by the academic editor and reviewer(s). You should upload this letter as a separate file labeled 'Response to Reviewers'.
- A marked-up copy of your manuscript that highlights changes made to the original version. You should upload this as a separate file labeled 'Revised Manuscript with Track Changes'.
- An unmarked version of your revised paper without tracked changes. You should upload this as a separate file labeled 'Manuscript'.

If you would like to make changes to your financial disclosure, please include your updated statement in your cover letter. Guidelines for resubmitting your figure files are available below the reviewer comments at the end of this letter.

If applicable, we recommend that you deposit your laboratory protocols in protocols.io to enhance the reproducibility of your results. Protocols.io assigns your protocol its own identifier (DOI) so that it can be cited independently in the future. For instructions see: https://journals.plos.org/plosone/s/submission-guidelines#loc-laboratory-protocols. Additionally, PLOS ONE offers an option for publishing peer-reviewed Lab Protocol articles, which describe protocols hosted on protocols.io. Read more information on sharing protocols at https://plos.org/protocols?utm_medium=editorial-email&utm_source=authorletters&utm_campaign=protocols.

We look forward to receiving your revised manuscript.

Kind regards,

Renier Mendoza

Academic Editor

PLOS One

Journal Requirements:

1. If the reviewer comments include a recommendation to cite specific previously published works, please review and evaluate these publications to determine whether they are relevant and should be cited. There is no requirement to cite these works unless the editor has indicated otherwise.

Additional Editor Comments:

A reviewer notes that only a few comments were addressed and that the manuscript requires substantial revision, particularly in rewriting the introduction to clearly motivate the study, situate it within existing literature, and explicitly state its novelty. Major issues remain with coherence, methodology clarity, placement of limitations and implications, correctness of references, and the quality and accuracy of figures, captions, and results discussion.

[Note: HTML markup is below. Please do not edit.]

Reviewer's Responses to Questions

**Comments to the Author**

1. If the authors have adequately addressed your comments raised in a previous round of review and you feel that this manuscript is now acceptable for publication, you may indicate that here to bypass the “Comments to the Author” section, enter your conflict of interest statement in the “Confidential to Editor” section, and submit your "Accept" recommendation.

Reviewer #3: (No Response)

Reviewer #4: (No Response)

**********

2. Is the manuscript technically sound, and do the data support the conclusions?

The manuscript must describe a technically sound piece of scientific research with data that supports the conclusions. Experiments must have been conducted rigorously, with appropriate controls, replication, and sample sizes. The conclusions must be drawn appropriately based on the data presented.

Reviewer #3: (No Response)

Reviewer #4: Partly

**********

3. Has the statistical analysis been performed appropriately and rigorously?

Reviewer #3: (No Response)

Reviewer #4: I Don't Know

**********

4. Have the authors made all data underlying the findings in their manuscript fully available?

The PLOS Data policy requires authors to make all data underlying the findings described in their manuscript fully available without restriction, with rare exception (please refer to the Data Availability Statement in the manuscript PDF file). The data should be provided as part of the manuscript or its supporting information, or deposited to a public repository. For example, in addition to summary statistics, the data points behind means, medians and variance measures should be available. If there are restrictions on publicly sharing data—e.g. participant privacy or use of data from a third party—those must be specified.

Reviewer #3: (No Response)

Reviewer #4: Yes

**********

5. Is the manuscript presented in an intelligible fashion and written in standard English?

PLOS ONE does not copyedit accepted manuscripts, so the language in submitted articles must be clear, correct, and unambiguous. Any typographical or grammatical errors should be corrected at revision, so please note any specific errors here.

Reviewer #3: (No Response)

Reviewer #4: No

**********

6. Review Comments to the Author

Please use the space provided to explain your answers to the questions above. You may also include additional comments for the author, including concerns about dual publication, research ethics, or publication ethics. (Please upload your review as an attachment if it exceeds 20,000 characters)

Reviewer #3: (No Response)

Reviewer #4: Only a few of the reviewers’ comments have been addressed by the authors.

1. The introduction needs to be rewritten.

- The authors should clearly state the significance and background of the study. Why is automated dispensing needed now? Why focus on antibiotic packaging? Using terms like urgent and critical does not sufficiently motivate the study’s aims.

- The related literature on image-based identification methods and the use of AI in pharmaceuticals is insufficient. Although references are provided, the authors should briefly discuss the findings of those studies.

- The novelty of the study is not highlighted in the introduction; it should not be placed in the results discussion. Is the novelty in the method itself, or in its application to antibiotic packaging?

2. There is no coherent flow of discussion between sections or within the sections.

3. The methodology section requires major revision.

- Consider adding a figure showing the overall workflow or algorithm at the beginning of the section. This will help readers follow the subsections more easily.

- The discussion on PAR should be detailed in Subsection 2.3.1 rather than in the results section.

- K-means clustering is mentioned but not explained; only the analysis is provided.

- The novelty of the DEA approach is not emphasized, and the discussion of DEA remains unclear and lacking in detail.

- Overall, the authors should introduce technical terms with sufficient context and explain the purpose of each algorithm before presenting it.

4. The study’s limitations should appear before Section 3.

5. The discussion of forward-looking implications belongs in the introduction.

6. Incorrect references were provided in response #8.

7. Figure captions have improved but still lack clarity. The discussion of results also needs improvement. It may be better to move the tables in the supplementary materials to the main text, as they are easier to understand than the figures in Section 3.

8. Figure 5 contains typographical errors.

9. Regarding comment #22, it is better to use the original figures and discuss each one.

**********

7. PLOS authors have the option to publish the peer review history of their article (what does this mean?). If published, this will include your full peer review and any attached files.

If you choose “no”, your identity will remain anonymous but your review may still be made public.

**Do you want your identity to be public for this peer review?**  For information about this choice, including consent withdrawal, please see our Privacy Policy.

Reviewer #3: No

Reviewer #4: No

**********

[NOTE: If reviewer comments were submitted as an attachment file, they will be attached to this email and accessible via the submission site. Please log into your account, locate the manuscript record, and check for the action link "View Attachments". If this link does not appear, there are no attachment files.]

To ensure your figures meet our technical requirements, please review our figure guidelines: https://journals.plos.org/plosone/s/figures

You may also use PLOS’s free figure tool, NAAS, to help you prepare publication quality figures: https://journals.plos.org/plosone/s/figures#loc-tools-for-figure-preparation.

NAAS will assess whether your figures meet our technical requirements by comparing each figure against our figure specifications.

# Author comment: Author response to Decision Letter 3

**DOI:** 10.1371/journal.pone.0354277.r006

31 Dec 2025

Manuscript ID: PONE-D-25-36293R2

Manuscript Title: Image-Based Identification and DEA-Based Optimization Modeling of

Antibiotic Packaging Using Unsupervised Learning Techniques

Journal: PLOS ONE

Dear Academic Editor,

We sincerely thank you and the reviewers for the careful evaluation of our manuscript and

for the constructive feedback provided. In response, we have undertaken a substantial

revision of the manuscript to address all comments raised, with particular attention to

conceptual clarity, methodological coherence, and alignment with PLOS ONE editorial

expectations.

Summary of Major Revisions

1. The Introduction was fully rewritten using a problem-driven framing to clearly motivate

the need for packaging-level antibiotic identification within automated pharmacy and

dispensing workflows, with explicit articulation of study novelty.

2. The Methods section was reorganized to clarify the purpose of each analytical component

before technical implementation, improving coherence and interpretability.

3. Packaging Area Ratio (PAR) was reframed as a normalization and design-relevant metric

and relocated from the Results section to the Methods section, in accordance with reviewer

guidance.

4. The role of K-means clustering was clarified as exploratory and unsupervised, with

cluster descriptions explicitly presented as post-hoc interpretations. Data Envelopment

Analysis (DEA) was reframed as a decision-oriented evaluation and benchmarking layer

rather than a classifier.

5. Methodological assumptions and study limitations were relocated to appear before the

Results section, as explicitly requested by the reviewer.

6. Figures and captions were revised to improve clarity and prevent overinterpretation. In

particular, figures were refined to clearly distinguish exploratory analysis, efficiency

evaluation, and descriptive feature emphasis, and typographical errors were corrected.

A detailed, point-by-point response to all reviewer comments, including the exact location

of each revision in the manuscript, is provided in the accompanying Response to Reviewers

document. All changes are also visible in the revised manuscript with tracked changes.

We believe that these revisions fully address the reviewers’ concerns and substantially

strengthen the manuscript. We appreciate the opportunity to revise our work and

respectfully submit the revised manuscript for your reconsideration.

Sincerely,

Phakdee Sukpornsawan

First Author

Suthabordee Muongmee

Corresponding Author

Attachment Submitted filename: Response to Reviewers R2.docx

# Peer review: Decision Letter 3

**DOI:** 10.1371/journal.pone.0354277.r007

**Authors:** Renier Mendoza

22 Jan 2026

PONE-D-25-36293R3

Image-Based Identification and DEA-Based Optimization Modeling of Antibiotic Packaging Using Unsupervised Learning Techniques

PLOS One

Dear Dr. Muongmee,

Thank you for submitting your manuscript to PLOS ONE. After careful consideration, we feel that it has merit but does not fully meet PLOS ONE’s publication criteria as it currently stands. Therefore, we invite you to submit a revised version of the manuscript that addresses the points raised during the review process.

Kindly refer to the attached reviewer comments. Please revise the manuscript accordingly before it can be accepted for publication.

Please submit your revised manuscript by Mar 08 2026 11:59PM. If you will need more time than this to complete your revisions, please reply to this message or contact the journal office at plosone@plos.org. When you're ready to submit your revision, log on to https://www.editorialmanager.com/pone/ and select the 'Submissions Needing Revision' folder to locate your manuscript file.

Please include the following items when submitting your revised manuscript:

- A letter that responds to each point raised by the academic editor and reviewer(s). You should upload this letter as a separate file labeled 'Response to Reviewers'.
- A marked-up copy of your manuscript that highlights changes made to the original version. You should upload this as a separate file labeled 'Revised Manuscript with Track Changes'.
- An unmarked version of your revised paper without tracked changes. You should upload this as a separate file labeled 'Manuscript'.

-->If you would like to make changes to your financial disclosure, please include your updated statement in your cover letter. Guidelines for resubmitting your figure files are available below the reviewer comments at the end of this letter.

If applicable, we recommend that you deposit your laboratory protocols in protocols.io to enhance the reproducibility of your results. Protocols.io assigns your protocol its own identifier (DOI) so that it can be cited independently in the future. For instructions see: https://journals.plos.org/plosone/s/submission-guidelines#loc-laboratory-protocols. Additionally, PLOS ONE offers an option for publishing peer-reviewed Lab Protocol articles, which describe protocols hosted on protocols.io. Read more information on sharing protocols at https://plos.org/protocols?utm_medium=editorial-email&utm_source=authorletters&utm_campaign=protocols.

We look forward to receiving your revised manuscript.

Kind regards,

Renier Mendoza

Academic Editor

PLOS One

Journal Requirements:

If the reviewer comments include a recommendation to cite specific previously published works, please review and evaluate these publications to determine whether they are relevant and should be cited. There is no requirement to cite these works unless the editor has indicated otherwise.

Please review your reference list to ensure that it is complete and correct. If you have cited papers that have been retracted, please include the rationale for doing so in the manuscript text, or remove these references and replace them with relevant current references. Any changes to the reference list should be mentioned in the rebuttal letter that accompanies your revised manuscript. If you need to cite a retracted article, indicate the article’s retracted status in the References list and also include a citation and full reference for the retraction notice.

[Note: HTML markup is below. Please do not edit.]

Reviewers' comments:

Reviewer's Responses to Questions

**Comments to the Author**

1. If the authors have adequately addressed your comments raised in a previous round of review and you feel that this manuscript is now acceptable for publication, you may indicate that here to bypass the “Comments to the Author” section, enter your conflict of interest statement in the “Confidential to Editor” section, and submit your "Accept" recommendation.

Reviewer #4: (No Response)

**********

2. Is the manuscript technically sound, and do the data support the conclusions?

The manuscript must describe a technically sound piece of scientific research with data that supports the conclusions. Experiments must have been conducted rigorously, with appropriate controls, replication, and sample sizes. The conclusions must be drawn appropriately based on the data presented.

Reviewer #4: Partly

**********

3. Has the statistical analysis been performed appropriately and rigorously?

Reviewer #4: N/A

**********

4. Have the authors made all data underlying the findings in their manuscript fully available?

The PLOS Data policy requires authors to make all data underlying the findings described in their manuscript fully available without restriction, with rare exception (please refer to the Data Availability Statement in the manuscript PDF file). The data should be provided as part of the manuscript or its supporting information, or deposited to a public repository. For example, in addition to summary statistics, the data points behind means, medians and variance measures should be available. If there are restrictions on publicly sharing data—e.g. participant privacy or use of data from a third party—those must be specified.

Reviewer #4: Yes

**********

5. Is the manuscript presented in an intelligible fashion and written in standard English?

PLOS ONE does not copyedit accepted manuscripts, so the language in submitted articles must be clear, correct, and unambiguous. Any typographical or grammatical errors should be corrected at revision, so please note any specific errors here.

Reviewer #4: No

**********

6. Review Comments to the Author

Please use the space provided to explain your answers to the questions above. You may also include additional comments for the author, including concerns about dual publication, research ethics, or publication ethics. (Please upload your review as an attachment if it exceeds 20,000 characters)

Reviewer #4: (No Response)

**********

7. PLOS authors have the option to publish the peer review history of their article (what does this mean?). If published, this will include your full peer review and any attached files.

If you choose “no”, your identity will remain anonymous but your review may still be made public.

**Do you want your identity to be public for this peer review?**  For information about this choice, including consent withdrawal, please see our Privacy Policy.

Reviewer #4: No

**********

[NOTE: If reviewer comments were submitted as an attachment file, they will be attached to this email and accessible via the submission site. Please log into your account, locate the manuscript record, and check for the action link "View Attachments". If this link does not appear, there are no attachment files.]

To ensure your figures meet our technical requirements, please review our figure guidelines: https://journals.plos.org/plosone/s/figures

You may also use PLOS’s free figure tool, NAAS, to help you prepare publication quality figures: https://journals.plos.org/plosone/s/figures#loc-tools-for-figure-preparation.

NAAS will assess whether your figures meet our technical requirements by comparing each figure against our figure specifications.

Attachment Submitted filename: PLOS ONE review_.pdf

# Author comment: Author response to Decision Letter 4

**DOI:** 10.1371/journal.pone.0354277.r008

29 Jan 2026

Dear Dr. Mendoza,

Thank you very much for your continued time and careful consideration of our manuscript. We are sincerely grateful for your additional editorial guidance, which has been instrumental in ensuring that the manuscript fully aligns with the scope, standards, and publication criteria of PLOS ONE.

In response to your editorial guidance, we have made the following final clarifications to the manuscript:

1. Scope and intended use of the framework

We have further clarified that the proposed approach is intended as an analytical and benchmarking framework for evaluating packaging-level machine-readability, rather than as a deployable clinical or regulatory decision-support system. This clarification has been incorporated into the Discussion and Conclusion sections to prevent any potential overinterpretation of the study’s scope or claims.

2. Reproducibility and transparency

To reinforce PLOS ONE’s reproducibility standards, we have added an explicit statement confirming that all algorithms, variables, feature definitions, and analytical steps are fully described in the Methods section and Supplementary Materials. This ensures that the analytical workflow can be independently understood and reproduced by readers without reliance on proprietary or undisclosed resources.

3. Clarification of figure interpretation

We have carefully reviewed all figures and their captions to ensure that they are consistently framed as exploratory, descriptive, or evaluative in nature, rather than as performance- or accuracy-benchmarking results. The revised captions and corresponding text now more clearly articulate the intended interpretive role of each figure and maintain a clear distinction between the Results and Discussion sections.

All substantive comments raised by the reviewers in the previous round have been fully addressed in the revised manuscript. The present revision therefore focuses exclusively on editorial-level clarification and alignment with journal policy, without introducing new analyses or extending the original claims.

We sincerely appreciate your guidance throughout the review process and thank you again for the opportunity to revise the manuscript accordingly. We hope that the manuscript in its current form will now be suitable for further consideration for publication in PLOS ONE.

Kind regards,

Suthabordee Muongmee, Ph.D.

Corresponding Author

Faculty of Pharmaceutical Sciences, Burapha University, Thailand

Email: suthabordee@go.buu.ac.th

ORCID: https://orcid.org/0000-0003-4033-3371

Attachment Submitted filename: Response to Reviewers (Detailed Point-by-Point).pdf

# Editor report: Decision Letter 4

**DOI:** 10.1371/journal.pone.0354277.r009

**Authors:** Renier Mendoza

5 Feb 2026

PONE-D-25-36293R4

Image-Based Identification and DEA-Based Optimization Modeling of Antibiotic Packaging Using Unsupervised Learning Techniques

PLOS One

Dear Dr. Muongmee,

Thank you for submitting your manuscript to PLOS ONE. After careful consideration, we feel that it has merit but does not fully meet PLOS ONE’s publication criteria as it currently stands. Therefore, we invite you to submit a revised version of the manuscript that addresses the points raised during the review process.

Please submit your revised manuscript by Mar 22 2026 11:59PM. If you will need more time than this to complete your revisions, please reply to this message or contact the journal office at plosone@plos.org. When you're ready to submit your revision, log on to https://www.editorialmanager.com/pone/ and select the 'Submissions Needing Revision' folder to locate your manuscript file.

Please include the following items when submitting your revised manuscript:

- A letter that responds to each point raised by the academic editor and reviewer(s). You should upload this letter as a separate file labeled 'Response to Reviewers'.
- A marked-up copy of your manuscript that highlights changes made to the original version. You should upload this as a separate file labeled 'Revised Manuscript with Track Changes'.
- An unmarked version of your revised paper without tracked changes. You should upload this as a separate file labeled 'Manuscript'.

-->If you would like to make changes to your financial disclosure, please include your updated statement in your cover letter. Guidelines for resubmitting your figure files are available below the reviewer comments at the end of this letter.

If applicable, we recommend that you deposit your laboratory protocols in protocols.io to enhance the reproducibility of your results. Protocols.io assigns your protocol its own identifier (DOI) so that it can be cited independently in the future. For instructions see: https://journals.plos.org/plosone/s/submission-guidelines#loc-laboratory-protocols. Additionally, PLOS ONE offers an option for publishing peer-reviewed Lab Protocol articles, which describe protocols hosted on protocols.io. Read more information on sharing protocols at https://plos.org/protocols?utm_medium=editorial-email&utm_source=authorletters&utm_campaign=protocols.

We look forward to receiving your revised manuscript.

Kind regards,

Renier Mendoza

Academic Editor

PLOS One

Journal Requirements:

1. If the reviewer comments include a recommendation to cite specific previously published works, please review and evaluate these publications to determine whether they are relevant and should be cited. There is no requirement to cite these works unless the editor has indicated otherwise.

2. Please review your reference list to ensure that it is complete and correct. If you have cited papers that have been retracted, please include the rationale for doing so in the manuscript text, or remove these references and replace them with relevant current references. Any changes to the reference list should be mentioned in the rebuttal letter that accompanies your revised manuscript. If you need to cite a retracted article, indicate the article’s retracted status in the References list and also include a citation and full reference for the retraction notice.

Additional Editor Comments:

The reviewer notes that while the manuscript’s writing has improved, many revisions appear aimed at addressing reviewer comments rather than improving clarity for readers, resulting in confusing presentation and sections that read like rebuttal text rather than a cohesive paper. Although the study is considered interesting and potentially valuable, it contains numerous issues—including unclear explanations, missing definitions, inconsistent terminology, typographical errors, figure and table problems, and misplaced or redundant content—that need substantial correction. Please refer to the attached file for the detailed review.

[Note: HTML markup is below. Please do not edit.]

[NOTE: If reviewer comments were submitted as an attachment file, they will be attached to this email and accessible via the submission site. Please log into your account, locate the manuscript record, and check for the action link "View Attachments". If this link does not appear, there are no attachment files.]

To ensure your figures meet our technical requirements, please review our figure guidelines: https://journals.plos.org/plosone/s/figures

You may also use PLOS’s free figure tool, NAAS, to help you prepare publication quality figures: https://journals.plos.org/plosone/s/figures#loc-tools-for-figure-preparation.

NAAS will assess whether your figures meet our technical requirements by comparing each figure against our figure specifications.

# Author comment: Author response to Decision Letter 5

**DOI:** 10.1371/journal.pone.0354277.r010

7 Feb 2026

Dear Academic Editor and Reviewers,

We sincerely thank the Academic Editor and reviewers for their careful evaluation of our manuscript and for the constructive comments provided. We appreciate the opportunity to revise our work and have undertaken a comprehensive revision to address the concerns raised.

In this revision (R4), our primary objective was to improve clarity, narrative coherence, and reader accessibility, in line with the Academic Editor’s observation that portions of the previous revision appeared rebuttal-driven rather than manuscript-centered. Importantly, no new data, experiments, or analytical outcomes were added. All revisions focus on restructuring, clarification, and alignment of presentation with PLOS ONE’s editorial expectations.

Below, we summarize how the major concerns were addressed.

1. Overall clarity and manuscript coherence

Comment: The manuscript shows improvement in writing but remains confusing in places, with sections reading like rebuttal text rather than a cohesive paper.

Response: The manuscript was restructured to ensure a reader-first narrative throughout. The Introduction was rewritten to align reader expectations with the analytical scope and aims of the study (Section 1). The Methods, Results, Discussion, and Conclusion sections were revised to clearly separate descriptive reporting from interpretation and to eliminate rebuttal-style phrasing. Each section now serves a distinct and non-overlapping role, resulting in a continuous and cohesive narrative intended for readers unfamiliar with the prior review history.

2. Methods presentation and role clarification

Comment: Explanations in the Methods section were unclear, with missing definitions and blurred methodological roles.

Response: The Methods section was rewritten for clarity and explicit role separation (Section 2). The sequential analytical logic of the study is now clearly described, and the function of each analytical component is delineated. In particular, Data Envelopment Analysis (DEA) is explicitly framed as a comparative evaluation layer rather than a predictive or classification model (Section 2.6.2). Definitions of key variables and metrics were standardized and streamlined, with detailed mathematical descriptions retained in the Supplementary Materials to preserve reproducibility while improving readability.

3. Terminology consistency and definitions

Comment: Inconsistent terminology and missing definitions reduced clarity.

Response: Terminology was standardized across the manuscript. Key concepts, metrics, and analytical components are now defined once and used consistently throughout all sections. Redundant or ambiguous terms were removed to reduce reader confusion.

4. Figures and tables

Comment: Several figures and tables were unclear, redundant, or misplaced, with captions that did not adequately support standalone interpretation.

Response: All figures and tables were systematically reviewed and revised (Figures 1–7, Table 1). Each figure was assigned a single narrative role—methods illustration, results visualization, or discussion synthesis—and captions were rewritten to be fully self-contained. Interpretive or performance-based language was removed from captions, and figure placement was adjusted to align with the revised manuscript structure.

5. Results reporting versus interpretation

Comment: Results and interpretation were intermingled, and some sections read as justificatory.

Response: The Results section was rewritten to present observed patterns and quantitative outcomes only, without interpretation or explanatory language. All interpretive discussion has been relocated to the Discussion section, ensuring a clear separation between reporting and interpretation.

6. Discussion tone and scope

Comment: The Discussion contained defensive language and overlapping content.

Response: The Discussion was rewritten to focus on balanced interpretation of the findings without justification of methodological choices or overstatement of impact (Section 4). Interpretations are now explicitly contextualized within the analytical scope of the study, and limitations are acknowledged in a neutral and transparent manner.

7. Conclusion and Abstract alignment

Comment: The manuscript would benefit from clearer synthesis and alignment across sections.

Response: The Conclusion was rewritten to provide a concise synthesis of the study’s analytical contribution without repeating results or extending claims beyond the study scope. The Abstract was carefully reviewed and retained without substantive modification, as it already accurately reflected the revised manuscript and maintained a descriptive, non-promotional tone consistent with PLOS ONE guidelines.

We believe these revisions improve the clarity and coherence of the manuscript and align it more closely with PLOS ONE’s editorial expectations, while preserving the integrity of the original study. We are grateful for the insightful feedback provided and appreciate the opportunity to revise our work.

Thank you for your time and consideration.

Sincerely,

Suthabordee Muongmee, Ph.D

Attachment Submitted filename: Response_R4_map to_Track_Changes.docx

# Peer review: Decision Letter 5

**DOI:** 10.1371/journal.pone.0354277.r011

**Authors:** Renier Mendoza

6 Apr 2026

PONE-D-25-36293R5

Image-Based Identification and DEA-Based Optimization Modeling of Antibiotic Packaging Using Unsupervised Learning Techniques

PLOS One

Dear Dr. Muongmee,

Thank you for submitting your manuscript to PLOS ONE. After careful consideration, we feel that it has merit but does not fully meet PLOS ONE’s publication criteria as it currently stands. Therefore, we invite you to submit a revised version of the manuscript that addresses the points raised during the review process.

The manuscript has improved in writing quality and presents interesting results, but its revisions appear focused on addressing reviewer comments rather than improving clarity, resulting in a presentation that is often confusing, inconsistent, and assumes too much prior knowledge. Numerous issues remain, including unclear explanations, missing definitions, typographical errors, and poorly presented figures and sections, indicating that substantial revisions are still needed for readability and coherence. Please refer to the attached file.

Please submit your revised manuscript by May 21 2026 11:59PM. If you will need more time than this to complete your revisions, please reply to this message or contact the journal office at plosone@plos.org. When you're ready to submit your revision, log on to https://www.editorialmanager.com/pone/ and select the 'Submissions Needing Revision' folder to locate your manuscript file.

Please include the following items when submitting your revised manuscript:

- A letter that responds to each point raised by the academic editor and reviewer(s). You should upload this letter as a separate file labeled 'Response to Reviewers'.
- A marked-up copy of your manuscript that highlights changes made to the original version. You should upload this as a separate file labeled 'Revised Manuscript with Track Changes'.
- An unmarked version of your revised paper without tracked changes. You should upload this as a separate file labeled 'Manuscript'.

If you would like to make changes to your financial disclosure, please include your updated statement in your cover letter. Guidelines for resubmitting your figure files are available below the reviewer comments at the end of this letter.

If applicable, we recommend that you deposit your laboratory protocols in protocols.io to enhance the reproducibility of your results. Protocols.io assigns your protocol its own identifier (DOI) so that it can be cited independently in the future. For instructions see: https://journals.plos.org/plosone/s/submission-guidelines#loc-laboratory-protocols. Additionally, PLOS ONE offers an option for publishing peer-reviewed Lab Protocol articles, which describe protocols hosted on protocols.io. Read more information on sharing protocols at https://plos.org/protocols?utm_medium=editorial-email&utm_source=authorletters&utm_campaign=protocols.

As the corresponding author, your ORCID iD is verified in the submission system and will appear in the published article. PLOS supports the use of ORCID, and we encourage all coauthors to register for an ORCID iD and use it as well. Please encourage your coauthors to verify their ORCID iD within the submission system before final acceptance, as unverified ORCID iDs will not appear in the published article. *Only*  the individual author can complete the verification step; PLOS staff *cannot*  verify ORCID iDs on behalf of authors.

We look forward to receiving your revised manuscript.

Kind regards,

Renier Mendoza

Academic Editor

PLOS One

**Journal Requirements:**

1. If the reviewer comments include a recommendation to cite specific previously published works, please review and evaluate these publications to determine whether they are relevant and should be cited. There is no requirement to cite these works unless the editor has indicated otherwise.

2. Please review your reference list to ensure that it is complete and correct. If you have cited papers that have been retracted, please include the rationale for doing so in the manuscript text, or remove these references and replace them with relevant current references. Any changes to the reference list should be mentioned in the rebuttal letter that accompanies your revised manuscript. If you need to cite a retracted article, indicate the article’s retracted status in the References list and also include a citation and full reference for the retraction notice.

[Note: HTML markup is below. Please do not edit.]

Reviewers' comments:

Reviewer's Responses to Questions

**Comments to the Author**

1. If the authors have adequately addressed your comments raised in a previous round of review and you feel that this manuscript is now acceptable for publication, you may indicate that here to bypass the “Comments to the Author” section, enter your conflict of interest statement in the “Confidential to Editor” section, and submit your "Accept" recommendation.

Reviewer #4: (No Response)

**********

2. Is the manuscript technically sound, and do the data support the conclusions?

The manuscript must describe a technically sound piece of scientific research with data that supports the conclusions. Experiments must have been conducted rigorously, with appropriate controls, replication, and sample sizes. The conclusions must be drawn appropriately based on the data presented.

Reviewer #4: Partly

**********

3. Has the statistical analysis been performed appropriately and rigorously?

Reviewer #4: No

**********

4. Have the authors made all data underlying the findings in their manuscript fully available?

The PLOS Data policy requires authors to make all data underlying the findings described in their manuscript fully available without restriction, with rare exception (please refer to the Data Availability Statement in the manuscript PDF file). The data should be provided as part of the manuscript or its supporting information, or deposited to a public repository. For example, in addition to summary statistics, the data points behind means, medians and variance measures should be available. If there are restrictions on publicly sharing data—e.g. participant privacy or use of data from a third party—those must be specified.

Reviewer #4: Yes

**********

5. Is the manuscript presented in an intelligible fashion and written in standard English?

PLOS ONE does not copyedit accepted manuscripts, so the language in submitted articles must be clear, correct, and unambiguous. Any typographical or grammatical errors should be corrected at revision, so please note any specific errors here.

Reviewer #4: Yes

**********

6. Review Comments to the Author

Please use the space provided to explain your answers to the questions above. You may also include additional comments for the author, including concerns about dual publication, research ethics, or publication ethics. (Please upload your review as an attachment if it exceeds 20,000 characters)

Reviewer #4: The manuscript’s readability has improved significantly. However, not all of the reviewer comments have been fully addressed (see red comments). In some cases, the authors appear to have removed portions of the methodological discussion rather than providing further clarification, which limits the transparency of the approach.

Additionally, reproducibility remains a concern, as the figures have not been revised to enhance clarity and understanding. The presence of typographical errors in the figures is also distracting and should be carefully corrected.

**********

7. PLOS authors have the option to publish the peer review history of their article (what does this mean?). If published, this will include your full peer review and any attached files.

If you choose “no”, your identity will remain anonymous but your review may still be made public.

**Do you want your identity to be public for this peer review?**  For information about this choice, including consent withdrawal, please see our Privacy Policy.

Reviewer #4: No

**********

[NOTE: If reviewer comments were submitted as an attachment file, they will be attached to this email and accessible via the submission site. Please log into your account, locate the manuscript record, and check for the action link "View Attachments". If this link does not appear, there are no attachment files.]

To ensure your figures meet our technical requirements, please review our figure guidelines: https://journals.plos.org/plosone/s/figures

You may also use PLOS’s free figure tool, NAAS, to help you prepare publication quality figures: https://journals.plos.org/plosone/s/figures#loc-tools-for-figure-preparation.

NAAS will assess whether your figures meet our technical requirements by comparing each figure against our figure specifications.

Attachment Submitted filename: PLOS ONE review__.pdf

# Author comment: Author response to Decision Letter 6

**DOI:** 10.1371/journal.pone.0354277.r012

11 Apr 2026

We thank the Academic Editor and Reviewer #4 for their constructive comments. We have revised the manuscript accordingly to improve clarity, consistency, readability, figure presentation, and methodological transparency.

A detailed point-by-point response is provided in the separate “Response to Reviewers” file. Revised versions of the manuscript with and without track changes have also been uploaded.

We hope the revised manuscript is now suitable for further consideration.

Attachment Submitted filename: Response to Reviewers (PLOS ONE)_2026.pdf

# Peer review: Decision Letter 6

**DOI:** 10.1371/journal.pone.0354277.r013

**Authors:** Renier Mendoza

19 Jun 2026

PONE-D-25-36293R6

Image-Based Identification and DEA-Based Optimization Modeling of Antibiotic Packaging Using Unsupervised Learning Techniques

PLOS One

Dear Dr. Muongmee,

Thank you for submitting your manuscript to PLOS ONE. After careful consideration, we feel that it has merit but does not fully meet PLOS ONE’s publication criteria as it currently stands. Therefore, we invite you to submit a revised version of the manuscript that addresses the points raised during the review process.

Please submit your revised manuscript by Aug 03 2026 11:59PM. If you will need more time than this to complete your revisions, please reply to this message or contact the journal office at plosone@plos.org. When you're ready to submit your revision, log on to https://www.editorialmanager.com/pone/ and select the 'Submissions Needing Revision' folder to locate your manuscript file.

Please include the following items when submitting your revised manuscript:

- A letter that responds to each point raised by the academic editor and reviewer(s). You should upload this letter as a separate file labeled 'Response to Reviewers'.
- A marked-up copy of your manuscript that highlights changes made to the original version. You should upload this as a separate file labeled 'Revised Manuscript with Track Changes'.
- An unmarked version of your revised paper without tracked changes. You should upload this as a separate file labeled 'Manuscript'.

If you would like to make changes to your financial disclosure, please include your updated statement in your cover letter. Guidelines for resubmitting your figure files are available below the reviewer comments at the end of this letter.

If applicable, we recommend that you deposit your laboratory protocols in protocols.io to enhance the reproducibility of your results. Protocols.io assigns your protocol its own identifier (DOI) so that it can be cited independently in the future. For instructions see: https://journals.plos.org/plosone/s/submission-guidelines#loc-laboratory-protocols. Additionally, PLOS ONE offers an option for publishing peer-reviewed Lab Protocol articles, which describe protocols hosted on protocols.io. Read more information on sharing protocols at https://plos.org/protocols?utm_medium=editorial-email&utm_source=authorletters&utm_campaign=protocols.

As the corresponding author, your ORCID iD is verified in the submission system and will appear in the published article. PLOS supports the use of ORCID, and we encourage all coauthors to register for an ORCID iD and use it as well. Please encourage your coauthors to verify their ORCID iD within the submission system before final acceptance, as unverified ORCID iDs will not appear in the published article. *Only*  the individual author can complete the verification step; PLOS staff *cannot*  verify ORCID iDs on behalf of authors.

We look forward to receiving your revised manuscript.

Kind regards,

Renier Mendoza

Academic Editor

PLOS One

Journal Requirements:

1. If the reviewer comments include a recommendation to cite specific previously published works, please review and evaluate these publications to determine whether they are relevant and should be cited. There is no requirement to cite these works unless the editor has indicated otherwise.

2. Please review your reference list to ensure that it is complete and correct. If you have cited papers that have been retracted, please include the rationale for doing so in the manuscript text, or remove these references and replace them with relevant current references. Any changes to the reference list should be mentioned in the rebuttal letter that accompanies your revised manuscript. If you need to cite a retracted article, indicate the article’s retracted status in the References list and also include a citation and full reference for the retraction notice.

Additional Editor Comments:

Kindly address the reviewer's remaining minor comments before the paper can be recommended for publication.

[Note: HTML markup is below. Please do not edit.]

Reviewer's Responses to Questions

**Comments to the Author**

1. If the authors have adequately addressed your comments raised in a previous round of review and you feel that this manuscript is now acceptable for publication, you may indicate that here to bypass the “Comments to the Author” section, enter your conflict of interest statement in the “Confidential to Editor” section, and submit your "Accept" recommendation.

Reviewer #4: (No Response)

**********

2. Is the manuscript technically sound, and do the data support the conclusions?

The manuscript must describe a technically sound piece of scientific research with data that supports the conclusions. Experiments must have been conducted rigorously, with appropriate controls, replication, and sample sizes. The conclusions must be drawn appropriately based on the data presented.

Reviewer #4: Yes

**********

3. Has the statistical analysis been performed appropriately and rigorously?

Reviewer #4: N/A

**********

4. Have the authors made all data underlying the findings in their manuscript fully available?

The PLOS Data policy requires authors to make all data underlying the findings described in their manuscript fully available without restriction, with rare exception (please refer to the Data Availability Statement in the manuscript PDF file). The data should be provided as part of the manuscript or its supporting information, or deposited to a public repository. For example, in addition to summary statistics, the data points behind means, medians and variance measures should be available. If there are restrictions on publicly sharing data—e.g. participant privacy or use of data from a third party—those must be specified.

Reviewer #4: Yes

**********

5. Is the manuscript presented in an intelligible fashion and written in standard English?

PLOS ONE does not copyedit accepted manuscripts, so the language in submitted articles must be clear, correct, and unambiguous. Any typographical or grammatical errors should be corrected at revision, so please note any specific errors here.

Reviewer #4: Yes

**********

6. Review Comments to the Author

Please use the space provided to explain your answers to the questions above. You may also include additional comments for the author, including concerns about dual publication, research ethics, or publication ethics. (Please upload your review as an attachment if it exceeds 20,000 characters)

Reviewer #4: I am still checking the same comments I raised in Revision 3 of the manuscript. Almost all of the comments have been addressed, but Figures 4 and 5 still contain inconsistencies that the authors should address. Otherwise, reproducibility remains an issue that needs to be resolved.

30. Figure 4B lacks a summary for Cluster 8. Additionally, there is no discussion/comments in the main text that talks about why there is NO Cluster 8 in their results. The authors said that they have addressed this but they still give the same figure as the one in Revision 3. NOT OK

31. Figure 4A uses different colors for clusters but does not include a legend identifying them. In particular, why are there blue, yellow, and green points in that plot. NOT OK

32. Figures 4 STILL contains unnecessary commentary since they still have the same Figure 4. NOT OK

33. Figure 5C and the new DEA efficiency summary does not match, e.g., Orcaptir has 0.93 score in the new figure but 0.96 in the old figure. No explanation for this in the rebuttal letter. Also, Cluster 2 – Orcaptrir Strip has 1.0 score but in the next table it is in Cluster 3 with score 0.93. The discussion said that efficiency scores ranged from 0.56 to 1.0 but it is not reflected in the figure. The caption for Figure 5C is Summary of DEA efficiency scores across representative packaging clusters and so, I do not know why there are no Clusters 5-9 and why is Cluster 4 repeated. Also, why the title DEA efficiency summary is repeated in the column and it is again repeated in the bottom of the figure.

**********

7. PLOS authors have the option to publish the peer review history of their article (what does this mean?). If published, this will include your full peer review and any attached files.

If you choose “no”, your identity will remain anonymous but your review may still be made public.

**Do you want your identity to be public for this peer review?**  For information about this choice, including consent withdrawal, please see our Privacy Policy.

Reviewer #4: No

**********

[NOTE: If reviewer comments were submitted as an attachment file, they will be attached to this email and accessible via the submission site. Please log into your account, locate the manuscript record, and check for the action link "View Attachments". If this link does not appear, there are no attachment files.]

To ensure your figures meet our technical requirements, please review our figure guidelines: https://journals.plos.org/plosone/s/figures

You may also use PLOS’s free figure tool, NAAS, to help you prepare publication quality figures: https://journals.plos.org/plosone/s/figures#loc-tools-for-figure-preparation.

NAAS will assess whether your figures meet our technical requirements by comparing each figure against our figure specifications.

Attachment Submitted filename: PLOS ONE review_6.pdf

# Author comment: Author response to Decision Letter 7

**DOI:** 10.1371/journal.pone.0354277.r014

26 Jun 2026

Dear Editor,

Thank you for your message. We have addressed the requested issues to allow the manuscript to proceed through the technical check.

First, we have uploaded a point-by-point Response to Reviewers document addressing all comments raised during this revision round. The manuscript has been revised accordingly, and all changes are shown in the marked-up manuscript file.

Second, we confirm that the submission contains the minimal data set underlying the findings of the study as Supporting Information. The dataset includes the raw values behind the reported descriptive statistics, the values used to generate graphs, extracted image-derived variables, annotated OCR results, clustering variables and assignments, and DEA input/output data required to reproduce the reported findings in their entirety. The Data Availability Statement has also been revised accordingly.

We hope that the revised submission now meets the technical requirements and can be forwarded for editorial consideration.

Sincerely,

Suthabordee Muongmee

Attachment Submitted filename: Response to Reviewer_R6.docx

# Editor report: Decision Letter 7

**DOI:** 10.1371/journal.pone.0354277.r015

**Authors:** Renier Mendoza

6 Jul 2026

Image-Based Identification and DEA-Based Optimization Modeling of Antibiotic Packaging Using Unsupervised Learning Techniques

PONE-D-25-36293R7

Dear Dr. Muongmee,

We’re pleased to inform you that your manuscript has been judged scientifically suitable for publication and will be formally accepted for publication once it meets all outstanding technical requirements.

Within one week, you’ll receive an e-mail detailing the required amendments. When these have been addressed, you’ll receive a formal acceptance letter and your manuscript will be scheduled for publication.

An invoice will be generated when your article is formally accepted. Please note, if your institution has a publishing partnership with PLOS and your article meets the relevant criteria, all or part of your publication costs will be covered. Please make sure your user information is up-to-date by logging into Editorial Manager at Editorial Manager® and clicking the ‘Update My Information' link at the top of the page. For questions related to billing, please contact billing support.

If your institution or institutions have a press office, please notify them about your upcoming paper to help maximize its impact. If they’ll be preparing press materials, please inform our press team as soon as possible -- no later than 48 hours after receiving the formal acceptance. Your manuscript will remain under strict press embargo until 2 pm Eastern Time on the date of publication. For more information, please contact onepress@plos.org.

Kind regards,

Renier Mendoza

Academic Editor

PLOS One

Additional Editor Comments (optional):

Reviewers' comments:

# Editor report: Acceptance letter

**DOI:** 10.1371/journal.pone.0354277.r016

**Authors:** Renier Mendoza

PONE-D-25-36293R7

PLOS One

Dear Dr. Muongmee,

I'm pleased to inform you that your manuscript has been deemed suitable for publication in PLOS One. Congratulations! Your manuscript is now being handed over to our production team.

At this stage, our production department will prepare your paper for publication. This includes ensuring the following:

* All references, tables, and figures are properly cited

* All relevant supporting information is included in the manuscript submission,

* There are no issues that prevent the paper from being properly typeset

You will receive further instructions from the production team, including instructions on how to review your proof when it is ready. Please keep in mind that we are working through a large volume of accepted articles, so please give us a few days to review your paper and let you know the next and final steps.

Lastly, if your institution or institutions have a press office, please let them know about your upcoming paper now to help maximize its impact. If they'll be preparing press materials, please inform our press team within the next 48 hours. Your manuscript will remain under strict press embargo until 2 pm Eastern Time on the date of publication. For more information, please contact onepress@plos.org.

You will receive an invoice from PLOS for your publication fee after your manuscript has reached the completed accept phase. If you receive an email requesting payment before acceptance or for any other service, this may be a phishing scheme. Learn how to identify phishing emails and protect your accounts at https://explore.plos.org/phishing.

If we can help with anything else, please email us at customercare@plos.org.

Thank you for submitting your work to PLOS ONE and supporting open access.

Kind regards,

PLOS ONE Editorial Office Staff

on behalf of

Dr. Renier Mendoza

Academic Editor

PLOS One
